# Genome-Wide Analysis of Immune Activation in Human T and B Cells Reveals Distinct Classes of Alternatively Spliced Genes

**DOI:** 10.1371/journal.pone.0007906

**Published:** 2009-11-19

**Authors:** Yevgeniy A. Grigoryev, Sunil M. Kurian, Aleksey A. Nakorchevskiy, John P. Burke, Daniel Campbell, Steve R. Head, Jun Deng, Aaron B. Kantor, John R. Yates, Daniel R. Salomon

**Affiliations:** 1 Department of Molecular & Experimental Medicine, The Scripps Research Institute, La Jolla, California, United States of America; 2 Department of Chemical Physiology, The Scripps Research Institute, La Jolla, California, United States of America; 3 Biotique Systems, Inc., Reno, Nevada, United States of America; 4 DNA Microarray Core, The Scripps Research Institute, La Jolla, California, United States of America; 5 Biomarker Discovery Sciences, PPD, Menlo Park, California, United States of America; 6 Scripps Center for Organ and Cell Transplantation, La Jolla, California, United States of America; Centre de Regulació Genòmica, Spain

## Abstract

Alternative splicing of pre-mRNA is a mechanism that increases the protein diversity of a single gene by differential exon inclusion/exclusion during post-transcriptional processing. While alternative splicing is established to occur during lymphocyte activation, little is known about the role it plays during the immune response. Our study is among the first reports of a systematic genome-wide analysis of activated human T and B lymphocytes using whole exon DNA microarrays integrating alternative splicing and differential gene expression. Purified human CD2^+^ T or CD19^+^ B cells were activated using protocols to model the early events in post-transplant allograft immunity and sampled as a function of time during the process of immune activation. Here we show that 3 distinct classes of alternatively spliced and/or differentially expressed genes change in an ordered manner as a function of immune activation. We mapped our results to function-based canonical pathways and demonstrated that some are populated by only one class of genes, like integrin signaling, while other pathways, such as purine metabolism and T cell receptor signaling, are populated by all three classes of genes. Our studies augment the current view of T and B cell activation in immunity that has been based exclusively upon differential gene expression by providing evidence for a large number of molecular networks populated as a function of time and activation by alternatively spliced genes, many of which are constitutively expressed.

## Introduction

The technology for investigating gene expression in cells and tissues has developed significantly over the last decade, making global gene expression profiling using microarrays relatively straightforward. However, until recently, the field has concentrated largely on studies of differential gene expression and discovering signatures that correlate with various biological challenges or disease states. Unfortunately, the premise of analyzing differential gene expression is limited by the view that molecular mechanisms or biomarkers are represented by classes of genes either up- or down-regulated in a given situation. Alternative splicing (AS) is a process by which a single pre-mRNA transcript can give rise to multiple protein isoforms through the mechanism of coordinated intron removal and differential exon joining. AS is a major source of diversity in the human proteome; as many as 75% [1], [2] of all human genes are alternatively spliced and the most recent study using next-generation sequencing technology indicates that 92–94% of human genes undergo alternative splicing [3], [4]. Splicing can modulate protein function by changing functional domains, affinities for assembly of heteromeric complexes, or altering mRNA stability. The advent of high-throughput genomics has dramatically changed the view of alternative splicing from a single gene perspective to the level of genome-wide discovery and quantification.

A major challenge in medicine is a molecular understanding of the immune response in conditions that range from autoimmune diseases such as Type I diabetes mellitus and multiple sclerosis to organ and cell transplantation. While there has been a steady progress in unraveling the complexities of innate and adaptive cellular immunity, there remain many unknowns particularly in our understanding of immune regulation and signaling networks that shape the course and outcome of lymphocyte activation. There are many reports of genome-wide differential gene expression of lymphocytes [5]–[27]. In contrast, literature for genome-wide alternative splicing in any system is still limited and, while AS is established to occur during lymphocyte activation, little is known about the role AS plays in immunity [25], [28]–[32]. One advance is the Affymetrix Human Exon 1.0 ST arrays that allow a high-throughput, genome-wide approach to analyze both differential gene expression and alternative splicing on a single chip. Human Exon 1.0 ST arrays have specific oligonucleotide probes for essentially every known and predicted exon in the latest build of the human genome. A number of publications exist for the use of these arrays in genome-wide gene expression analysis [33]–[45], four of which included analysis of alternative splicing [34], [35], [46], [47] in the context of cancer cells. However, presently there are only a few papers on activation-dependent genome-wide alternative splicing in human T lymphocytes, using the Agilent 44K microarray platform that allows monitoring of >5000 cassette-type AS events in human cells [28], as well as Exon 1.0 ST array to study the genome-wide effect of silencing a splicing factor during T cell activation [48].

The present study is among the first to report a systematic genome-wide analysis integrating alternative splicing and differential gene expression with analysis of functional molecular networks populated during human lymphocyte activation. We used the Human Exon 1.0 ST array platform to integrate the analysis of genome-wide gene expression with alternative splicing as a function of time during activation of purified human CD2+ T cells or purified CD19+ B cells. We detected 3 distinct classes of genes in both T and B cells that changed as a function of immune activation: 1) differentially expressed and alternatively spliced, 2) constitutively expressed and alternatively spliced, and 3) differentially expressed without alternative splicing.

Approximately 60% or more of all the alternatively spliced genes in T cells are differentially expressed at the mRNA transcript level during activation and only 25–30% of B cell transcripts. However, analysis of constitutively expressed transcripts demonstrated that 50–70% in T cells and about 40% in B cells are alternatively spliced. These results demonstrate the significant increase in transcriptional diversity generated by alternative splicing during lymphocyte activation and emphasize how large the relative contribution to this diversity is made by alternative splicing of constitutively expressed genes. Finally, we mapped the three classes of genes to functional molecular networks. These studies expand the current view of T and B cell activation in immunity that has been based exclusively upon differential gene expression by providing evidence for a large number of molecular networks populated as a function of time and activation by alternatively spliced genes.

## Results

### Differential Gene Expression and Alternative Splicing in Activated Lymphocytes

We wanted to identify changes in global transcript expression and alternative splicing (AS) as a function of activation of primary human T and B lymphocytes. We purified T and B cells using magnetic bead-coupled CD2 and CD19 antibodies, respectively. T cells were activated using anti-CD3/CD28 beads and B cells were activated with anti-CD40 antibody cross-linked by anti-IgG plus rIL2 and rIL10. We confirmed cell activation by looking at known activation marker expression with multi-channel parallel FACS at 0 and 48 hours post activation. T48 hours was selected for activation marker analysis from previous observations that showed the peak of activation by 48 hours (data not shown). For T cells, many differences were observed between stimulated and control T cells: after activation for 48 hours, activation markers were higher, chemokine receptors were low, cytokine producing cells were higher and many other marked changes were present, representative of T cell activation. Higher levels (relative intensity) of activation markers on CD4+ T cells were observed for CD134 (OX40), CD150 (SLAM), CD25 (IL-2Rα), CD69 (early activation marker), and CD71 (transferin receptor) at 48 hours compared to unactivated cells. Also, a higher fraction of stimulated CD4+ T cells were positive for these activation markers: CD134 (78%), CD150 (50%), CD25 (90%), CD69 (95%), and CD71 (80%). We also observed higher levels of these activation markers on stimulated CD8+ T cells and higher fraction of stimulated CD8+ T cells were positive for these same activation markers. We also detected lower levels of chemokine receptors CCR5, CD183 (CXCR3), and CD197 (CCR7) on stimulated CD4+ and CD8+ T cells compared to resting. Both CD4+ and CD8+ T cells expressed lower levels of CD127 (IL-7 receptor α chain) after stimulation. We also observed much higher intensities for such cytokines as IFNγ, IL-2 and TNFα intensities on stimulated CD4+ T cells compared to control CD4+ T cells, where they were almost undetectable. A higher fraction of stimulated CD4+ T cells produced IFNγ, IL-2, and TNFα as determined by intracellular cytokine staining while no detectable IL-10 or IL-4 producing cells were detected in either control or stimulated CD4+ T cells. We observed similar results for stimulated CD8+ T cells compared to resting CD8+ T cells. (**See Figure S1, Table S1 and Table S2**).

B cells stimulated for 48 hours showed some levels (mean channel intensity above 200) of positive staining for activation markers CD150, CD25, and CD69, whereas resting B cells do not express these markers (mean channel intensity<100). Stimulated B cells showed significantly higher levels of positive staining for co-stimulatory molecules CD54, CD80, CD86 compared to resting B cells. Resting B cells are typically positive for CD21 and CD62L whereas we only observed 40% of stimulated B cells that were positive for these markers consistent with the fact that CD21 and CD62L are shed during B cell activation. (**Figure S2 and Table S1 and Table S2**).

Total RNA and protein was extracted at 0, 24, 48 and 72 hours post activation. Both differential gene expression and alternative splicing were detected using Affymetrix Human Exon 1.0 ST Arrays that are comprised of different probes designed to interrogate all the known exon sequences in the current version of the human genome. The Exon array results were analyzed using the bioinformatics tool, XRAY, which identifies the alternative use of specific exons in each gene detected between multiple time points.

As shown in **Table 1**, the total number of detected AS genes is significantly higher at all time points in T cells compared to B cells. This is also true for the subset of genes that are annotated as alternatively spliced in the public domain of RefSeq. Based on a parallel analysis of mRNA transcript levels and alternative exon usage, we observed two classes of AS genes: 1) differentially expressed (AS+DE) and, 2) constitutively expressed (AS+CE). We also identified a separate third class of genes that are differentially expressed (DE) but show no evidence of alternative splicing.

**Table 1:** Detectable differential gene expression and alternative splicing changes in activated lymphocytes.

Cell type	Hours post activation	Alternatively spliced genes (% genes detected)	Alternatively spliced genes with differential gene expression (% total differentially expressed)	Alternatively spliced genes with constitutive gene expression (% total constitutively expressed)	Differentially expressed genes with no alternative splicing (% up-regulated)	Constitutively expressed genes (% genes detected)
**T**	24	3,863 (54%)	2161 (46%)	1702 (73%)	2,473 (86%)	2328 (33%)
**T**	48	3,154 (55%)	1394 (44%)	1760 (69%)	1,761 (88%)	2547 (45%)
**T**	72	1,584 (45%)	703 (41%)	881 (49%)	1,027 (83%)	1783 (51%)
**B**	24	991 (47%)	222 (63%)	769 (44%)	131 (92%)	1747 (83%)
**B**	48	967 (42%)	260 (46%)	707 (41%)	310 (87%)	1722 (75%)
**B**	72	973 (44%)	96 (41%)	877 (44%)	140 (79%)	1990 (89%)

Next, taking all the annotated, AS genes, we compared the relative numbers of constitutively and differentially expressed transcripts. The point of this comparison is that activation-dependent changes in cell function can be caused by AS even if the transcript's absolute expression is unchanged. During T cell activation, there are comparable numbers of AS+DE and AS+CE genes. In contrast, the opposite is true for B cell activation in which the number of AS+CE genes is consistently higher. Finally, the majority of differentially expressed genes in both T and B cell activation are up-regulated (79% to 92%; **Table 1**).

### Alternatively Spliced Genes in T Cell Activation Are Either Differentially or Constitutively Expressed

The first class of AS genes comprise those that are both alternatively spliced and differentially expressed (AS+DE). First, the total numbers of AS+DE genes at 24, 48 and 72 hours are significantly greater during T cell activation (2161, 1394, 703, respectively) as compared to B cell activation (222, 260, 95, respectively) (**Table 1**). Second, in these primary activated human T cells approximately 60% or more of all the alternatively spliced genes are also differentially expressed at the mRNA transcript level at all points during the course of activation. These results contrast sharply with data from an analysis of AS in an activated human T cell line, Jurkat, that found that the majority of AS genes do not demonstrate differential expression [28]. The difference could be due to the choice of cells (primary T cells vs. Jurkat), the activation method (anti-CD28/CD3 vs. PMA) or the technology (Agilent 44K AS arrays).

**Figure 1:**
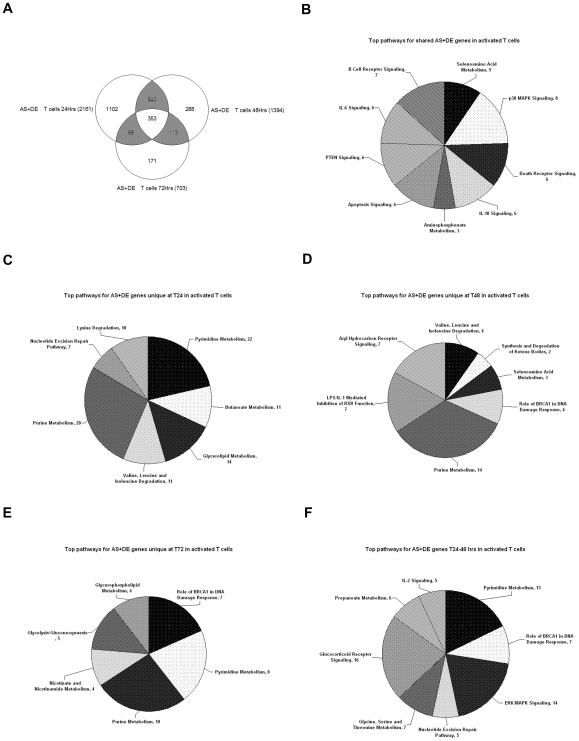
Canonical pathways for alternatively spliced and differentially expressed T cell genes (p<0.001) shows both immune/inflammatory mechanisms and metabolism. (A) Venn diagram representing the alternatively spliced and differentially expressed (AS+DE) genes detected at 24, 48, and 72 hours in activated T cells, with total number of detected genes per time point shown in parenthesis, number of genes unique to each time point shown by the number inside the circle and number of genes shared between time points in the shaded overlapping areas, with number of genes detected at all three time points shown in the central overlap. (B–F) Pie charts representing the Ingenuity Pathway Analysis output for the top canonical pathways populated by AS+DE genes during T cell activation: (B) shared at all time points, (C) genes unique to 24 hour time-point, (D) genes unique to the 48 hour time-point, (E) genes unique to the 72 hour time-point, (F) genes uniquely detected during the transition between 24 and 48 hour time-points. Numbers next to pathway name indicate the number of detected genes that are assigned to each pathway. The size of each shaded slice is proportional to the number of genes per pathway compared to total number of genes per chart.

There are 353 AS+DE genes common to all 3 activation time points in T cells, which we propose are genes involved in the entire process of T cell activation (**Figure 1a**). Six of the top ten canonical pathways identified with Ingenuity Pathway Analysis (IPA) are signaling via IL-6, IL-10, p38 MAPK, T cell receptors, death receptors and apoptosis (**Figure 1b**). Next, we analyzed genes detected as AS+DE but unique to each time point (1102, 288 and 171 genes at 24, 48 and 72 hours, respectively), which we propose are time-specific drivers of activation. Surprisingly, the canonical pathways identified at all three time points are largely involved in cell metabolism rather than pathways typically linked to immunity (**Figure 1c–e**). On the other hand, activation is a cellular process, whether it is driven by immunity, inflammation or any other mechanism. The most populated pathway is purine metabolism present at all three time points (28, 14, and 10 genes at 24, 48 and 72 hours, respectively). It is important to emphasize that these represent 52 different genes sequentially expressed and alternatively spliced as a function of time in a single pathway.

Finally, there is third subset of AS+DE genes that are shared between two activation times (640 at 24 and 48 hours) and (113 at 48 and 72 hours), which we propose are genes that are involved in the transitions between events in activation marked by the specific time points (**Figure 1a**). Pathway analysis of these shared AS+DE genes revealed both immune/inflammatory mechanisms such as ERK/MAPK, glucocorticoid receptor and IL-2 signaling and metabolic pathways such as pyrimidine, propanoate and glycine, serine and threonine metabolism (**Figure 1f**). In effect, the canonical functions of these AS+DE genes that are shared between the transition time points are a mix of the elements found in the AS+DE genes unique or common to all time points.

**Figure 2:**
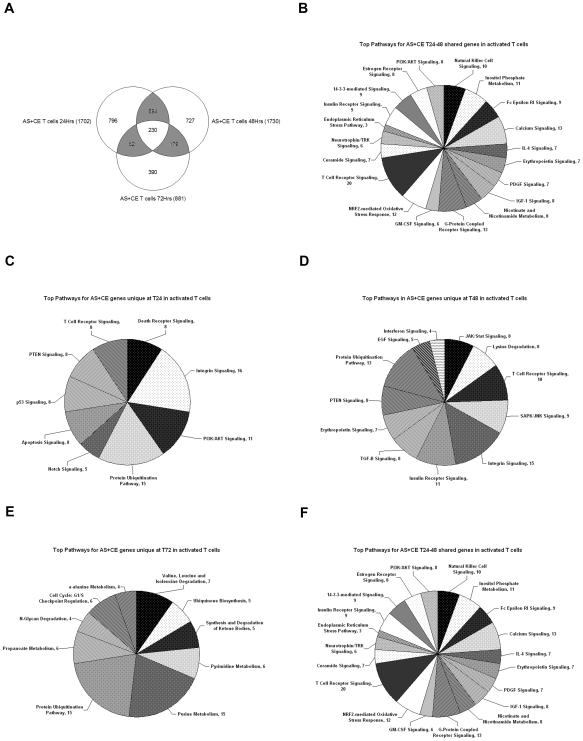
Alternatively spliced/constitutively expressed T cell genes (p<0.001) unique to each activation time-point populate canonical pathways for immune/inflammation. (A) Venn diagram representing the alternatively spliced/constitutively expressed (AS+CE) genes detected at 24, 48, and 72 hours in activated T cells, with total number of detected genes per time point shown in parenthesis, number of genes unique to each time point shown by the number inside the circle and number of genes shared between time points in the shaded overlapping areas, with number of genes detected at all three time points shown in the central overlap. (B–F) Pie charts representing the Ingenuity Pathway Analysis output for the top canonical pathways populated by AS+CE genes during T cell activation: (B) shared at all time points, (C) genes unique to 24 hour time-point, (D) genes unique to the 48 hour time-point, (E) genes unique to the 72 hour time-point, (F) genes uniquely detected during the transition between 24 and 48 hour time-points. Numbers next to pathway name indicate the number of detected genes that are assigned to each pathway. The size of each shaded slice is proportional to the number of genes per pathway compared to total number of genes per chart.

The second class of AS genes comprise those that are alternatively spliced but constitutively expressed (AS+CE) as a function of activation (**Table 1**). For example, from a total of 2,328 constitutively expressed genes at 24 hours, we detected 1702 that were alternatively spliced (73%). This is remarkable because the typical approach to gene expression profiling experiments is to exclude the constitutively expressed genes from further analysis. While 230 AS+CE genes were shared at all three time points after activation, functional pathway analysis did not reveal any specific themes (**Figure 2a**). In contrast, analysis of the AS+CE genes unique to the three time points revealed a prevalence of canonical pathways linked to immune/inflammation including T cell receptor, PI3K/AKT, integrin and death receptor/apoptosis signaling at 24 hours and JAK/Stat, SAPK/JNK, TGFβ, and interferon signaling at 48 hours (**Figure 2c–e**). The third subset of AS+CE genes are those shared between the transition time points. For example, for 24 vs. 48 hours, there are 594 AS+CE genes identified that are linked to a large number of canonical pathways associated with immune/inflammatory activation including signaling by IL-4, GM-CSF, insulin, IGF-1, PDGF, and via PI3K/AKT (**Figure 2f**).

A literature search for genes that are experimentally validated to be AS in T cells or hematopoietic lineage cells under any conditions revealed only 41 genes (**Table S3**). At 24 hours, we detected 21 of the known AS genes, 17 of which were called as AS by XRAY analysis. At 48 hours, we detected expression 25 known AS genes, 13 of which were called as AS (p<0.001; **Table 2**) and 17 of which were shared with the known genes identified at 24 hours (data not shown). Seven of these 25 genes were also detected in activated B cells at 48 hours. Finally, at 72 hours, we detected expression of 13 known AS genes, 6 of which were called as AS by XRAY analysis. Overall, we detected 29 of the 41 known AS genes in the literature (71%) during the course of activating T cells.

**Table 2:** Detected known T cell alternatively spliced genes at 48 hrs post activation.

Gene Symbol	Gene Annotation	Max. fold- change	Differential gene expression ANOVA P value (FDR corrected)	Alternative exon usage ANOVA P value (FDR corrected)
ILF3	interleukin enhancer binding factor 3 90 kDa	1.21	2.96E-01	2.42E-15
MAP4K4	mitogen-activated protein kinase kinase kinase kinase 4	−1.21	8.57E-02	1.81E-12
CD44*	CD44 molecule (Indian blood group)	1.01	1.00E+00	8.52E-09
FKBP2*	FK506 binding protein 2 13 kDa	2.01	4.65E-05	1.53E-07
MAP4K1	mitogen-activated protein kinase kinase kinase kinase 1	1.31	1.19E-01	1.05E-06
CUGBP2	CUG triplet repeat RNA binding protein 2	−1.11	1.00E+00	1.13E-06
FKBP8	FK506 binding protein 8 38 kDa	1.81	1.09E-03	1.72E-06
IRAK1	interleukin-1 receptor-associated kinase 1	1.31	2.42E-04	1.68E-05
FKBP11	FK506 binding protein 11 19 kDa	−1.11	1.00E+00	5.31E-05
FKBP1A	FK506 binding protein 1A 12 kDa	1.91	3.31E-06	6.24E-05
LEF1	lymphoid enhancer-binding factor 1	−1.51	6.03E-01	1.50E-04
ICAM1	intercellular adhesion molecule 1 (CD54) human rhinovirus receptor	2.01	6.64E-06	1.55E-04
FKBP3*	FK506 binding protein 3 25 kDa	1.81	1.69E-03	8.08E-04
IRF1	interferon regulatory factor1	−1.81	5.12E-03	1.01E-03
FYN	FYN oncogene related to SRC FGR YES	1.61	8.50E-02	2.51E-03
CD79B	CD79b molecule immunoglobulin-associated β	1.11	1.00E+00	2.83E-03
GATA3	GATA binding protein 3	1.31	7.69E-05	1.43E-02
CLK2	CDC-like kinase 2	1.71	2.08E-03	9.38E-02
SYK	spleen tyrosine kinase	−1.11	1.01E-04	1.20E-01
IL2	interleukin 2	7.31	1.24E-08	1.00E+00
HRB*	HIV-1 Rev binding protein	2.91	4.40E-06	1.00E+00
HMMR*	hyaluronan-mediated motility receptor (RHAMM)	3.11	4.59E-09	1.00E+00
HIF1A*	hypoxia-inducible factor 1 alpha subunit (basic helix-loop-helix transcription factor)	3.61	2.39E-06	1.00E+00
FKBP4*	FK506 binding protein 4 59 kDa	4.41	8.70E-07	1.00E+00
CTLA4	cytotoxic T-lymphocyte-associated protein 4	7.41	1.32E-08	1.00E+00

* Also detected in B cells at 48 hours, with FKBP4 and HRB predicted to be alternatively spliced and HMMR and FKBP2 to be also differentially up-regulated. See supplemental **Table S1** for complete list of known AS genes.

Given the large number of AS genes identified by our analysis at 48 hours in activated T cells (1394 AS+DE and 1730 AS+CE; **Table 1**), we analyzed the gene expression of known or putative splicing factors at the same time point. Of 25 splicing factors expressed in activated T cells at 48 hours, 15 showed significant differential gene expression (p<0.001) and 11 were alternatively spliced (p<0.001; **Table S4**). Interestingly, the majority of splicing factors that are alternatively spliced are not differentially expressed (80%). In agreement with prior observations [28], [49], the majority of splicing factors with significant differential gene expression were up-regulated by activation. The same analysis revealed 11 known splice factors in activated B cells at 48 hours, 10 of which were also detected in T cells with the same expression patterns (**Table S4**).

### Genes That Are Differentially Expressed but Not Alternatively Spliced during T Cell Activation

**Figure 3:**
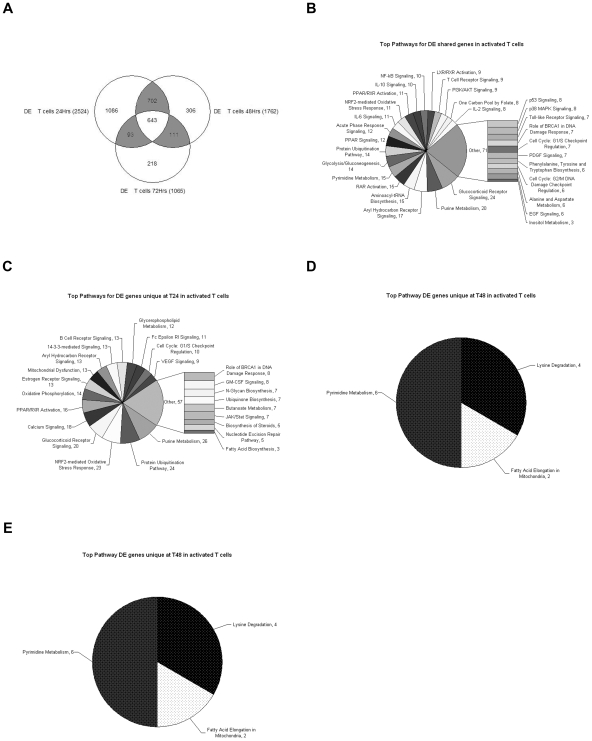
T cell genes differentially expressed (p<0.001) but without splicing that are common to all time-points populate a mix of immune/inflammation and metabolic pathways. (A) Venn diagram representing the differentially expressed genes with no alternative splicing (DE) detected at 24, 48, and 72 hours in activated T cells, with total number of detected genes per time point shown in parenthesis, number of genes unique to each time point shown by the number inside the circle and number of genes shared between time points in the shaded overlapping areas, with number of genes detected at all three time points shown in the central overlap. (B–E) Pie charts representing the Ingenuity Pathway Analysis output for the top canonical pathways populated by DE genes during T cell activation: (B) shared at all time points, (C) genes unique to 24 hour time-point, (D) genes unique to the 48 hour time-point, (E) genes unique to the 72 hour time-point. Numbers next to pathway name indicate the number of detected genes that are assigned to each pathway. The size of each shaded slice is proportional to the number of genes per pathway compared to total number of genes per chart.

After T cell activation, there are 2524, 1762 and 1065 differentially expressed (DE) genes where no AS was detected at 24, 48 and 72 hours, respectively (**Figure 3a**). Of these, 643 genes are DE at all three time points. Functional analysis revealed a large number of canonical pathways linked to immune/inflammatory networks including signaling via T cell receptors, Toll-like receptors, PI3K/AKT, IL-6, IL-2, IL-10, p38 MAPK, PDGF, acute phase response and glucocorticoid receptors (**Figure 3b**). However, there are also multiple pathways representing metabolic networks including pyrimidine, purine, protein ubiquitination, glycolysis/gluconeogenesis, aryl hydrocarbon receptor signaling and aminoacyl-tRNA biosynthesis. There are 1086 genes uniquely DE at 24 hours (**Figure 3c**), the peak of gene expression for T cell activation. The four highest ranked pathways (>20 genes/pathway) are purine metabolism, protein ubiquitination, NRF2-mediated oxidative stress response and glucocorticoid receptor signaling. It is notable that between the DE genes unique at 24 hours and those DE genes common at all three time points, we can populate the canonical pathway for protein ubiquitination with 38 different genes, 46 for purine metabolism and 44 for glucocorticoid receptor signaling. In combination with the 52 AS+DE genes populating the purine metabolism pathway already described above, we have identified a total of 98 different genes in this single pathway of a total of 418 known genes in the pathway as listed in Ingenuity (23%).

### RT-PCR and RNA Deep Sequencing Validation of Predicted AS Events Confirms 19 New AS Gene Candidates in Activated T Cells

We ranked and filtered the entire list of AS candidate genes predicted by the XRAY analysis of our whole exon array data based on the FDR corrected p values determined by ANOVA for alternative probe set usage. From the list of candidates with statistically significant alternative probe set usage (p values<0.001), we then selected 32 candidate AS genes at random, not previously known in the literature to be alternatively spliced in activated T cells. The splicing events predicted in these 32 candidate genes included cassette exons, mutually exclusive exons, bleeding exons, intron retention, alternative start sites, and alternative UTRs (see **Table 3** and **Table S5** for primer and probe set information). Primers were designed to target flanking, constitutively expressed and RefSeq annotated exons. RT-PCR was performed for individual total RNA samples from 8 of the 10 donors used in the generation of the whole exon array data comparing the activation of T cells at T0 and T48 hours.

**Table 3:** Differentially alternatively spliced candidate genes selected from the Whole Exon Gene Array and RNA-Seq data for RT-PCR validation in activated T cells between 0 and 48 hours.

Gene	Probe Set ID	Probe Set exon	Expressed p-value^1^	AS PS p-value^2^	F^3^	R^3^	Alternative Splicing Event^4^	XRAY 1.0ST exon call (ΔT48/T0) 5	RNA Seq exon counts (ΔT48/T0, 3/3 donors) 6
TNFAIP3	2927516	Int4	4.56E-37	8.38E-04	3	5	intron Inclusion	Lower Splicing	Increased(3/3)
TA-NFKBH	3860131	Ex3	4.25E-08	4.71E-04	2	6	cassette exon	Higher Splicing	Not detected (3/3)
TA-NFKBH	3860129	Ex4	1.39E-07	2.60E-04	2	6	cassette exon/alt-3′	Higher Splicing	Decreased (2/3)
CDC42	2324660	Ex7	4.43E-29	9.89E-04	6	7/8	mutually exclusive exon	Higher Splicing	Decreased(1/3), Increased 2/3
HSPA14	3236409	Ex4	1.65E-07	2.74E-06	1	5	cassette exon	Higher Splicing	Decreased (2/3)
GZMB	3558395	Ex1	6.15E-27	6.24E-09	1	3	alternative 5′ UTR	Higher Splicing	Decreased (1/3) Increased (2/3)
CLASP1	2573699	Ex24	4.29E-06	2.85E-05	21	25	cassette exon	Higher Splicing	Undetected(1/3), Increased (2/3)
LYK5	3766291	Ex11ext	1.38E-37	1.36E-06	9	11b	mutually exclusive exon	Higher Splicing	Decreased (1/3), Increased (2/3)
LYK5	3766290	Ex11ext	1.16E-17	7.27E-04	9	11b	mutually exclusive exon	Higher Splicing	Decreased (1/3), Increased (2/3
CDCA5	3377433	Ex4	8.86E-42	1.68E-07	3	5	cassette exon	Lower Splicing	Not detected (3/3)
ILF17	2957120	Ex2	1.13E-24	1.98E-06	1	3	alternative 5′ UTR	Higher Splicing	Not detected (2/3), Increased (1/3)
CSF1	2351093	Ex6ext	2.53E-20	1.14E-06	5	8	mutually exclusive exon	Higher Splicing	Not detected (1/3), Increased (1/3), Decreased (1/3)
CREM	3242380	Ex8a	1.90E-29	5.85E-04	7	9	cassette exon	Lower Splicing	Not detected (1/3), Increased (1/3), Decreased (1/3)
WDR51A	2675975	E8	6.62E-10	4.74E-06	7	9b	cassette exon	Higher Splicing	Decreased (1/3), Undetected (2/3)
UNC45A	3608546	E10	2.03E-20	6.41E-05	9	11	cassette exon	Lower Splicing	Increased (1/3), Undetected (2/3)
TUBA1A	3453838	E4	1.05E-48	5.56E-08	4	4	alt-C-term	Higher Splicing	Decreased (3/3)
TUBA1A	3453839	E4	2.86E-59	1.25E-07	4	4	alt-C-term	Higher Splicing	Decreased (3/3)
TIMM50	3833104	E6	1.47E-34	1.07E-05	2	7	bleeding exon	Higher Splicing	Increased (2/3)
SLC25A3	3427827	E2	5.25E-24	2.62E-06	1c-2	4	cassette exon	Higher Splicing	Increased (1/3), Decreased (1/3), Undetected (1/3)
RUNX1	3930438	E1	9.21E-41	4.29E-05	1	3	bleeding exon	Lower Splicing	Decreased (2/3) Increased (1/3)
RUNX1	3930435	E1	3.09E-19	7.32E-10	1	3	bleeding exon	Lower Splicing	Decreased (2/3) Increased (1/3)
RUNX1	3930436	E1	2.61E-37	7.34E-08	1	3	bleeding exon	Lower Splicing	Decreased (2/3) Increased (1/3)
RBBP6	3653318	E1	1.14E-35	7.32E-05	1	5	cassette exon	Higher Splicing	Decreased (2/3)
RBBP6	3653319	E1	4.56E-25	1.57E-04	1	5	cassette exon	Higher Splicing	Decreased (2/3)
RBBP6	3653326	E3	6.47E-51	1.39E-04	1	5	cassette exon	Higher Splicing	Decreased (2/3)
RBBP6	3653335	E4	7.31E-23	4.56E-04	1	5	cassette exon	Higher Splicing	Not Detected
NLN	2812388	E9	4.35E-15	8.17E-04	8	10	alt 5′UTR/bleeding exon	Higher Splicing	Not Detected
NCAPD3	3399565	E29	5.19E-30	2.08E-08	28	30	cassette exon	Higher Splicing	Not Detected
NAB2	3417820	E2	2.13E-35	1.73E-05	2	5	alternate 5′ End	Lower Splicing	Increased (2/3)
KIF4A	3980562	E2	6.34E-25	7.40E-11	1	3	bleeding exon/retained intron	Higher Splicing	Increased (1/3), Undetected (2/3)
ING3	3021135	E5	1.72E-19	3.75E-06	4	5	alt-C-term/bleeding exon	Higher Splicing	Decreased (3/3)
CDC27	3760685	E1	7.49E-42	9.73E-08	1	3	alternative 5′ UTR	Higher Splicing	Increased (1/3), Undetected (2/3)
CDC27	3760684	E1	6.82E-21	9.58E-08	1	3	alternative 5′ UTR	Higher Splicing	Increased (1/3), Undetected (2/3)

1 ANOVA p-value for Probe Set expression as calculated by XRAY.

2 ANOVA p-value for alternative Probe Set usage (alternative splicing) as calculated by XRAY.

3 Exons in which the F (forward) and R (reverse) PCR primers are located.

4 Known Alternative Splicing Events for the detected Probe Set shown in the UCSC Genome browser.

5 Change in alternative splicing as calculated by XRAY analysis at 48 vs 0 hours (activation at 48 hours).

6 Change in exon counts (number of reads that align to the exon) as observed from the RNA-Seq data at T48 vs T0 hours. Abbreviations: Ex  =  exon, Int  =  Intron, Ext  =  extended exon, alt-C-term  =  alternative C terminal, alt-3′  =  alternate 3

**Figure 4:**
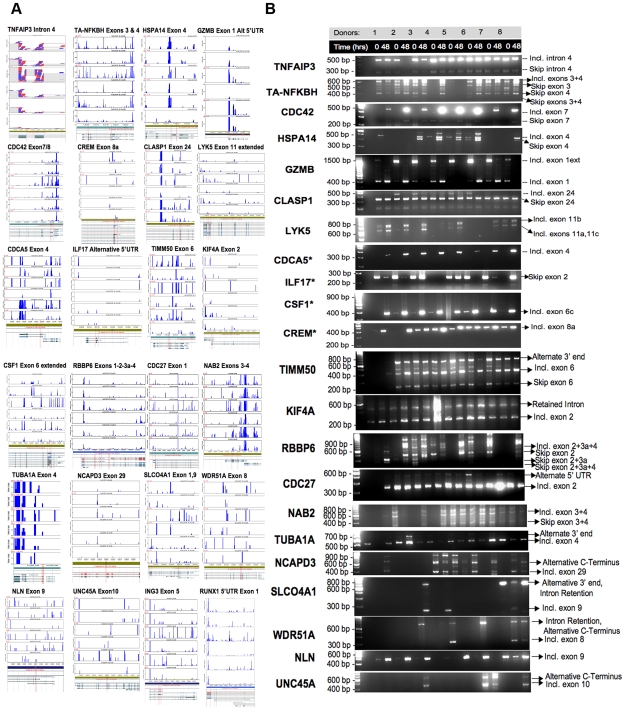
Validation of differentially alternatively spliced gene candidates in activated T cells between 0 and 48 hours. (A) GenomeStudio view of 24 of 32 total candidate AS genes, 17 validated by PCR and 7 candidates chosen as examples of those that were not PCR validated. Genomic stacked alignment plots with top 3 rows representing samples 3 to 1 (top to bottom) at T0 and bottom 3 rows representing samples 3 to 1 at T48, respectively. The y-axis represents the number of reads and the height of each bar is determined by calculating the overlapping sequence tags for each base-pair in a 4 pixel wide region and taking the maximum value. Splice junctions, when shown, are indicated by the grey dotted lines. Exons of interest are bordered by red vertical lines. Ensemble transcript splice variants are shown below for known annotated splice isoforms. (B) RT-PCR validation of 24 of 32 total candidate AS genes, 17 validated by PCR and 7 candidates chosen as examples of those that were not PCR validated. PCR was performed with primers to the neighboring constitutively expressed exons and the products were resolved on 2.5% agarose gels. Gene symbols are listed on the right, time points and donor for each sample are listed in the top 2 panels, and observations of splice events for indicated exons are given to the right (incl  =  exon retained, skip  =  exon spliced). Star next to a gene name indicates that the validation results did not show the predicted splice variants. 100 bp ladder is visualized in the left-most lane. Unmarked bands most likely represent minor splice variants, heteroduplexes or RT-PCR artifacts.

Alternative splicing was also tested in parallel by deep sequencing of total RNA from activated T cells of 3 donors at T0 and T48 hours using an Illumina GAIIx instrument. We analyzed the sequencing results using the GenomeStudio RNA Sequencing Module (Illumina, Inc., San Diego, CA) that also allowed us to visualize the aligned reads and analyze results of exon counts, genes and splice junctions found in our data. These data are shown in **Figure 4a** for 24 candidate genes. We totaled the exon counts in GenomeStudio for all the known exons of each of the 32 candidate genes for validation for 3 donors at T0 vs. T48 hours of activation. These results were then compared to XRAY whole exon array results and 19/32 (59%) candidate alternatively spliced genes were validated in at least one or more donor (**Table S5**).

The RT-PCR results validate distinct alternative splicing of the predicted isoforms for 17 of the 32 candidates (**Figure 4b** and **Table 3**). RT-PCR for alternatively splicing is known to be prone to artifacts [35], [47] with more bands than predicted appearing on the gel as seen for several of our candidates (e.g. TA-NFKBH, HSPA14). These unlabeled bands are thought to represent heteroduplexes of the two AS isoforms [35], [47]. We observed some donor-to-donor variability for HSPA14, CLASP1, LYK5, ILF17, NCAPD3, SLCO4A1, WDR51A, and UNC45A, which is to be expected, as alternative splicing is likely to fluctuate among different individuals due to the intrinsic variability in response to lymphocyte activation and potentially genetic differences. We were unable to validate the predicted splice isoforms for 15 of the 32 candidates (examples shown for CDCA5, ILF17, CSF1, CREM, NLN) because we failed to simultaneously detect both the spliced and unspliced variants required as the frame of reference necessary to validate the AS. For example, CSF1 demonstrates only a single PCR product at the molecular weight of the highly spliced exon 6c variant, which is nonetheless still consistent with the higher splicing predicted by XRAY for T48 hours (**Table 3**; **Figure 4**). For CDCA5 there is a single RT-PCR product at T48 hours consistent with the unspliced form of exon 4 also as predicted by XRAY. The deep sequencing data provides an additional set of data for reference and while limited to 3 donors, it confirms to some degree the splicing predictions of XRAY for 14 of the 32 candidate genes (**Table S5** and **Table 3**).

### Additional Validation of Alternative Splicing during T Cell Activation by Proteomics

We used tandem mass spectrometry proteomics (MudPIT; see Methods) to analyze and validate alternative splicing in T cells at 0 hrs and 48 hrs post activation. We detected a total of 1510 unique proteins. The full list was searched for proteins with multiple detected isoforms representing alternative splicing. We found 1091 proteins (72%) with 2 or more isoforms. Based on **Table 1**, the percentage of AS transcripts was 54% at 24 hours and 55% at 48 hours.

By matching protein and transcript identifications (i.e. proscripts), there are 190 AS+DE proscripts and 247 AS+CE proscripts (**Table S6**). We confirmed that multiple protein isoforms were detected in both these groups consistent with the alternative splicing predicted by the whole exon array data: 64/190 (34%) AS+DE and 119/247 (48%) AS+CE proscripts. We found that 2 of our 32 candidate genes for validation were proscripts detectable at both T0 and T48 and both were validated by the proteomic data (GZMB and CDC27). Of the remaining 1073 proteins that were not paired with whole exon array-detected transcripts, 825 (77%) had multiple isoforms detected by mass spectrometry. We also observed 317 proscripts that had between 2 and 9 different isoforms detected by proteomics but that were not predicted to be alternatively spliced by the whole exon array data.

### Canonical Pathways Populated by All Three Classes of Genes

**Figure 5:**
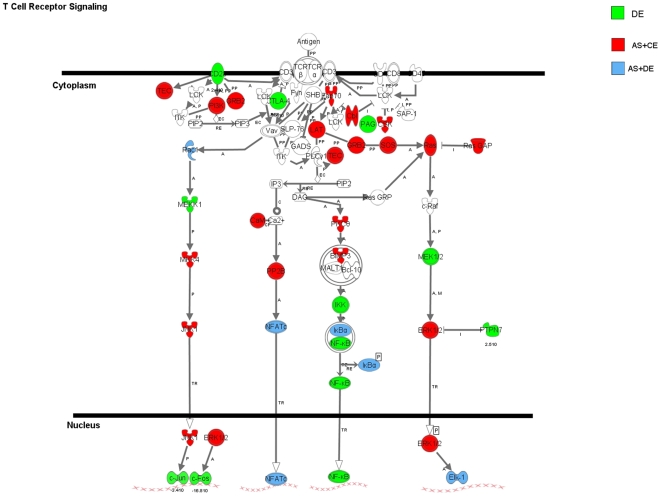
T cell receptor signaling pathway is populated by members from all three classes of genes during activation. Schematic diagram represents the T cell receptor signaling pathway populated by the Ingenuity Pathway Analysis using genes assigned to all three classes detected throughout the time course of activation. Shapes in green represent the class of genes with differential expression without alternative splicing (DE). Shapes in red represent the class of genes that are alternatively spliced with constitutive gene expression (AS+CE). Shapes in blue represent genes both alternatively spliced and differentially expressed (AS+DE). Legend for Ingenuity-Mapped Interactions: A  =  Activation, C  =  Causes/Leads to, CP  =  Chemical-Protein Interaction, EC  =  Enzyme Catalysis, I  =  Inhibition, M  =  Biochemical Modification, P  =  Phosphorylation/Dephosphorylation, PP  =  Protein-Protein binding, RE  =  Reaction, TR  =  Translocation. An arrow pointing from A to B signifies that A causes B to be activated.

In the context of all the different functional networks that we have discussed for T cell activation, we found a subset of canonical pathways that were populated as a function of time after activation by genes representing all three classes: AS+DE, AS+CE and DE, alone. As shown in **Table 4**, there are 9 pathways where these genes populate from 15 to 50% of the total known genes for that pathway. Using the top pathway, T cell receptor signaling, as an example, it is evident that every level of the signal pathway from the cell surface to the nucleus is represented (**Figure 5**).

**Table 4:** Top canonical pathways populated by genes representing all three classes.

Top Pathways	Populated genes/total in pathway (%)	T24 unique	T48 unique	T72 unique	Shared
**T Cell Receptor Signaling**	52/105 (49.5%)	13DE, 8AS+CE	10AS+CE	N/A	9DE, 7AS+DE, 5AS+CE
**Protein Ubiquitination Pathway**	81/205 (39.5%)	24DE, 15AS+CE	13AS+CE	15AS+CE	14DE
**Purine Metabolism**	121/418 (29%)	26DE, 28AS+DE	14AS+DE	10AS+DE, 15 AS+CE	20DE, 8AS+CE
**IL-10 Signaling**	16/73 (22%)	N/A	N/A	N/A	10DE, 6AS+DE
**Death Receptor Signaling**	14/66 (21%)	8AS+CE	N/A	N/A	6AS+DE
**IL-6 Signaling**	17/96 (17.7%)	N/A	N/A	N/A	11DE, 6AS+DE
**Glucocorticoid Receptor Signaling**	44/279 (16%)	20DE	N/A	N/A	24DE
**Integrin Signaling**	31/196 (16%)	16AS+CE	15AS+CE	N/A	N/A
**Apoptosis Signaling**	14/91 (15.3%)	8AS+CE	N/A	N/A	6AS+DE

### Functions of Alternatively Spliced and Differentially Expressed Genes Demonstrates Different Networks for T and B Cell Activation at 48 Hours Post Activation

While the major focus of this work has been on T cell activation, we compared the top canonical pathways identified in T cells for AS+DE, AS+CE and DE genes to those in B cells at the 48-hour time point. For this purpose we used the total genes comprising each class as shown in **Table 5**. While many T cells pathways are linked to known immune/inflammatory networks, the majority of the top B cell pathways are linked to cell growth, survival and metabolism. Nonetheless, several canonical pathways populated by all three classes of genes are shared between activated T and B cells that reflect their common lymphocyte lineage. These are genes linked to cell cycle G1/S checkpoint, (9 genes), G2/M DNA damage checkpoint regulation (7 genes), as well as signaling via aryl hydrocarbon receptor (15 genes), apoptosis (6 genes), death receptors (8 genes) and VEGF (3 genes).

**Table 5:** Top canonical pathway genes identified by Ingenuity Network analysis in activated lymphocytes at 48 hours.

**T Cells**		
**Differential gene expression with no alternative splicing**	**Differential gene expression with alternative splicing**	**Constitutive gene expression with alternative splicing**
Aryl Hydrocarbon Receptor Signaling (32)*	Apoptosis Signaling (19)	Actin Cytoskeleton Signaling (45)
Glycolysis/Gluconeogenesis (22)	B Cell Receptor Signaling (17)	ERK/MAPK Signaling (39)
IL-6 Signaling (18)	NF-kB Signaling (17)	Integrin Signaling (32)
PPAR Signaling (16)	SAPK/JNK Signaling 17)	SAPK/JNK Signaling (31)
Cell Cycle: G1/S Checkpoint Regulation (15)	p38 MAPK Signaling (14)	PI3K/AKT Signaling (31)
IL-10 Signaling (14)	IL-6 Signaling (13)	B Cell Receptor Signaling (29)
IGF-1 Signaling (14)	Death Receptor Signaling (11)	Apoptosis Signaling (26)
Cell Cycle: G2/M DNA Damage Checkpoint Regulation (13)	Cell Cycle: G1/S Checkpoint Regulation (8)	T Cell Receptor Signaling (20)
IL-2 Signaling (11)	IL-10 Signaling (8)	PTEN Signaling (19)
EGF Signaling (8)	Cell Cycle: G2/M DNA Damage Checkpoint Regulation (6)	Fc Epsilon RI Signaling (19)
		Natural Killer Cell Signaling (19)
		Neurotrophin/TRK Signaling (16)
		VEGF Signaling (16)
		IGF-1 Signaling (16)
		JAK/Stat Signaling (15)
		PDGF Signaling (15)
		TGF-B Signaling (15)
		GM-CSF Signaling (14)
		IL-4 Signaling (14)
		FGF Signaling (14)
		EGF Signaling (10)
		IL-2 Signaling (10)
**B Cells**		
**Differential gene expression with no alternative splicing**	**Differential gene expression with alternative splicing**	**Constitutive gene expression with alternative splicing**
NRF2-mediated Oxidative Stress Response (10)	Aryl Hydrocarbon Receptor Signaling (10)	Protein Ubiquitination Pathway (15)
Protein Ubiquitination Pathway (10)	Cell Cycle: G1/S Checkpoint Regulation (7)	VEGF Signaling (10)
Aryl Hydrocarbon Receptor Signaling (7)	p53 Signaling (6)	Citrate Cycle (7)
Cell Cycle: G2/M DNA Damage Checkpoint Regulation (5)	Death Receptor Signaling (5)	Death Receptor Signaling (7)
Aminoacyl-tRNA Biosynthesis (5)	Protein Ubiquitination Pathway (5)	Pyruvate Metabolism (7)
p53 Signaling (4)	Apoptosis Signaling (3)	Apoptosis Signaling (7)
Antigen Presentation Pathway (3)	Biosynthesis of Steroids (2)	Aryl Hydrocarbon Receptor Signaling (7)
Cell Cycle: G1/S Checkpoint Regulation (3)	Cell Cycle: G2/M DNA Damage Checkpoint Regulation (2)	p53 Signaling (5)
Apoptosis Signaling (2)	Aminoacyl-tRNA Biosynthesis (2)	Nucleotide Excision Repair Pathway (4)
	Endoplasmic Reticulum Stress Pathway (1)	Endoplasmic Reticulum Stress Pathway (3)

* Number of molecules in each pathway.

## Discussion

Despite many recent reports on alternative splicing, studies of genome-wide alternative splicing in the immune system are still limited and, while alternative splicing (AS) is established to occur during lymphocyte activation, little is known about the role AS plays or its impact on immunity. Alternative splicing of pre-mRNA is a mechanism that increases the protein repertoire of a single gene sequence by including or excluding exons during post-transcriptional processing. There is evidence in different model systems for alternative splicing of as many as 75% to 94% of all human genes [1], [2], [4]. Splicing can modulate protein function by changing functional domains, affinities for assembly of heteromeric complexes, or altering mRNA stability.

The majority of what we know about AS is based on conventional single-gene approaches though more recently new technologies to facilitate genome-wide studies have been used [2], [34], [35], [47], [50]–[54]. Our study is among the first reports of a systematic genome-wide analysis integrating AS and differential gene expression as a function of human lymphocyte activation. In this study, CD2^+^ T and CD19^+^ B lymphocytes from 10 normal human donors were activated and sampled sequentially for 72 hrs to model the early events in post-transplant allograft immunity. Our hypotheses were that: 1) AS plays an important role in regulating immunity, 2) these genes escape identification if only differential expression is considered, and 3) identification and mapping of AS genes to known molecular pathways is an important first step in understanding how alternative splicing regulates the immune response.

In this study, we have performed a quantitative analysis of alternatively spliced exons in activated T and B lymphocytes as a function of time after activation. Our activation protocols were chosen to model the events in post-transplantation immune responses where T cell activation occurs through co-stimulatory signaling [55]–[57] and B cell activation involves T cell-derived signals [12], [58]–[65]. We used Affymetrix Human Exon 1.0 ST arrays, a technology that permits a comprehensive and unbiased coverage of the whole transcriptome. This approach enabled us to perform two complementary levels of analysis: gene-level differential expression and exon-level analysis distinguishing between different alternatively spliced isoforms of a gene. Exon-level analysis on a whole-genome scale allows detection of specific splicing events and treats individual exons as independent objects to observe differential skipping or inclusion.

Our results (**Table 1**) indicate that there are three detectable classes of genes that change during the course of immune activation in our analysis: 1) alternatively spliced and differentially expressed (AS+DE), 2) alternatively spliced and constitutively expressed (AS+CE), and 3) genes that are differentially expressed but show no evidence of alternative splicing (DE). The majority of differentially expressed genes in both T and B cell activation are up-regulated. During T cell activation, we observed that there are comparable numbers of AS+DE and AS+CE genes. In contrast, the opposite is true for B cell activation in which the number of AS+CE genes is consistently higher. Also, the total number of detected AS genes is significantly higher at all time points in T cells compared to B cells. These results reflect the higher level of cellular complexity that characterize the T cells including CD4^+^ and CD8^+^ as well as functional T cell subsets such as Tregs and Th17.

We observed that in primary activated human T cells at 24 and 48 hours, more than 50% of all detected genes are alternatively spliced. However, of all the constitutively expressed genes, 73% are alternatively spliced at 24 hours. In contrast, of all the differentially expressed genes, only 46% are alternatively spliced at 24 hours. Clearly, a higher percentage of constitutively expressed genes in T cells are alternatively spliced. This is not what we would have expected. Our original assumption was that higher levels of differential gene expression would correlate with higher levels of alternative splicing. In contrast, our results suggest that in the evolution of cellular regulation a choice has been made such that one class of genes is primarily regulated by differential expression during activation while another class is regulated, despite constitutive expression, by alternatively splicing. Because the majority of constitutively expressed genes are alternatively spliced, there is the potential of a tremendous amount of functional gene regulation by alternatively splicing that has not been considered previously.

A different point is made by an analysis of the total number of differentially and constitutively expressed genes in activated T cells that are alternatively spliced. Because there are significantly more differentially expressed genes in this setting, our data demonstrates that approximately 60% or more of all the alternatively spliced genes are differentially expressed at the mRNA transcript level and this is true at all points during the course of activation. The opposite seems to be true in activated B cells, where 60% or more of all the alternatively spliced genes are constitutively expressed. Thus, it is clear that alternative splicing is still an important mechanism for generating complexity from the T cell's transcriptome of differentially expressed genes. Our results are in contrast to those of another recent publication in which the majority of alternatively spliced transcripts were not differentially expressed [28]. These data were generated with a different array technology and an activation protocol based on a potent cell mitogen. We believe that our use of primary human lymphocytes and two activation strategies specifically chosen to model natural immune activation via T cell co-stimulation (CD3/CD28) and T cell help for B cell activation (anti-CD40/IL2/IL10) is more representative of biology in this context.

During T cell activation, the AS+DE genes detected and shared at all time points are enriched in canonical pathways that reveal immune/inflammatory mechanisms such as signaling via IL-6, IL-10, p38 MAPK, T cell receptors, death receptors and apoptosis. These are all canonical pathways reflecting the functional agenda of T lymphocyte activation during an immune response. We also observed a subset of AS+DE genes unique to each time point and these genes populated canonical pathways representing mostly metabolic mechanisms, with purine metabolism being the highest populated pathway. These results are somewhat surprising and suggest that the course of T cell activation is also driven by regulation of selected cellular metabolic pathways. We also detected unique AS+DE genes that marked the time point transitions (e.g. 24 to 48 hours). We propose that these genes regulate the transitions between events occurring during activation as a function of time. Remarkably, pathway analysis of these transition point-specific genes revealed another set of genes that are only detected during transitions but further populate many of the pathways already identified and linked to both immune/inflammatory mechanisms and metabolism. We propose that specific molecular networks are established early in T cell activation and then additional and critical gene components of these pathways are added as a function of time to obtain and/or regulate the impact of these networks on the final result. As a specific example to support these findings, we have populated the known canonical pathway for T cell receptor signaling using the three classes of genes we identified here: AS+DE, AS+CE and DE, alone (**Figure 5**).

During T cell activation, AS+CE genes unique to each of the three time points populate canonical pathways linked to immune/inflammation including T cell receptor, PI3K/AKT, integrin and death receptor/apoptosis signaling at 24 hours and JAK/Stat, SAPK/JNK, TGFβ, and interferon signaling at 48 hours. Thus, it is evident that AS+CE genes add more members to populate the same canonical pathways linked to AS+DE genes but also contribute to additional pathways during activation. Similar to what we found for the AS+DE genes, AS+CE genes that mark the time transition points during T cell activation are linked to predominantly different networks including signaling by IL-4, GM-CSF, insulin, IGF-1, PDGF, and via PI3K/AKT.

We also observed the up-regulation of a large number of the known splicing factors. A number of splicing factors were also alternatively spliced, indicating a possibility of auto-regulatory mechanisms involved at the level of regulating splicing. Interestingly, the majority of splicing factors that are alternatively spliced are not differentially expressed. It is a common belief that cell activation is regulated predominantly by differential gene expression. This belief is why essentially every paper on profiling of cell transcriptomes during experimental or clinical events is concentrated on identifying patterns of differential gene expression and the pathways impacted by these changes. Thus, we thought that alternative splicing would also follow differential gene expression and genes associated with alternative splicing would be changing during activation. This is clearly not the case.

An important question is how well do the results of our 1.0ST Exon Arrays reflect the alternative splicing during B and T lymphocyte activation? The first important point is that the results are relatively consistent for each class of activated lymphocyte and each time point as reflected by the high statistical values we achieved for thousands of genes with 10 donors for each time point for each class of cells. The second point is that our conclusions for overall per cent alternatively spliced genes (50–70%) are well in line with the literature. A third point is that we used a tandem mass spectrometry proteomics approach to validate alternative splicing and found over 800 alternatively spliced proscripts. Finally, we chose at random a set of 32 candidate genes from the list of statistically significant candidates for alternative splicing for RT-PCR validation. We designed PCR primers to exons flanking the predicted sites of alternative splicing. From the 32 candidate genes chosen in activated T cells at 48 hours, we observed multiple splice variants for 17 candidate genes. The other candidates demonstrated only a single detectable PCR product and thus, could not be definitively validated. To put these validation results in a proper context, previous studies for alternative splicing validations done by PCR range from 33% to 86% success rates [66]. Thus, our validation success rate of 53% is within the expected range for this particular platform and method of validation.

The tandem mass spectrometry proteomic dataset identified 317 matching transcripts/proteins (called proscripts) for which there were multiple isoforms demonstrated consistent with alternative splicing. A functional network analysis using Ingenuity revealed 10 highly significant networks linked to mechanisms of cancer, cell cycle regulation, cellular assembly, molecular transport and cell metabolism. Several canonical pathways were highly significantly over-represented in activated T cells (**Table S7**) and included aminoacyl tRNA biosynthesis, purine metabolism, and integrin signaling, which were also identified by the whole exon array data analysis shown in **Tables 4** and **5**.

The final question is what kind of specific mechanistic insights can we obtain by analysis of alternative splicing during lymphocyte activation? For this purpose we discuss the potential impact of the alternative splicing we identified in this study for 5 genes. First, TNFAIP3 (tumor necrosis factor alpha-induced protein 3) localized in the nucleus, is rapidly induced by Tumor Necrosis Factor (TNF), and is critical for limiting inflammation by terminating TNF-induced NF-kappa B responses as well as TNF-mediated apoptosis. Our analysis of the whole exon array data, validated by PCR, indicated that the AS probe set coding for Intron 4 is preferentially spliced out at T0 compared to T48, resulting in intron retention at 48 hours (**Table 3**). The biological consequences induced by the retention of Intron 4, including the potential that this contributes to the protein sequence in this important regulatory Zn-finger protein, are presently unknown.

TA-NFKBH (T-cell activation NFKB-like protein) is localized in the nucleus and functions in the regulation of inflammatory responses through regulation of NF-kappa-B activity. It is also thought to regulate TCR-induced negative selection of thymocytes. We identified probe sets coding for two cassette exons, Exons 3 and 4, as alternatively spliced at 48 hours. Results of deep RNA sequencing at T48 indicated that Exon 3 is more commonly spliced out than Exon 4 and RT-PCR showed evidence of splicing either exon, but almost no splicing of both at the same time. These splicing events modify the 5′UTR of the mature transcript as well as changing part of the coding region. The coding region affected contains 6 Ankyrin repeats that mediate protein-protein interactions in very diverse families of proteins.

CDC42 (cell division cycle 42) is a small GTPase of the Rho-subfamily that regulates signaling pathways that control cell morphology, migration, endocytosis and cell cycle progression. Alternative splicing of this gene is known to produce multiple transcript variants. Our whole exon array data for T cell activation demonstrated alternative splicing of Exon 7, which is a mutually exclusive exon with Exon 8. This splicing changes a small part of the coding region as well as the entire 3′UTR altering the C-terminus of the translated protein. This observation was confirmed by PCR and the deep RNA sequencing data.

HSPA14 (heat shock 70 kDa protein 14) belongs to the family of heat shock hsp70 proteins required for key cellular processes and cell survival in response to environmental changes but this protein family is incompletely characterized at this time. Our results predict alternative splicing of Exon 4 at T48 hours of activation. This cassette exon is in the coding region and is predicted to have 6 motifs: N-glycosylation site, Protein kinase C phosphorylation site, Casein kinase II phosphorylation site, N-myristoylation site, Eukaryotic thiol (cysteine) proteases histidine active site, and a motif called heat shock hsp70 proteins family signature 3.

GZMB (granzyme 2, cytotoxic T-lymphocyte-associated serine esterase 1) is crucial for the mechanism of cell killing induced by cytotoxic T cells in cell-mediated immune responses. Our whole exon array data, PCR and deep RNA sequencing data indicated that the 5′UTR and part of the coding region of Exon 1 are alternatively spliced out during activation. Since the coding region contains the trypsin-like serine protease domain required for the function of granzyme B in cell killing, the alternative splicing demonstrated here might regulate the structure and activation capacity of the molecule as a function of T cell activation.

It is clear that the ability to identify alternative splicing at a genome-wide level adds another dimension to our view of lymphocyte activation. Exon arrays provide a platform for identifying such genome-wide changes normalizing for individual exon and gene expression between any two states in question. However, it is critical to emphasize that mechanistic understandings of alternative splicing events at this level will require additional tools to efficiently map thousands of alternatively spliced exons to predicted protein sequences and from sequences to functional and regulatory domains. Thus, one point of the present work is to provide proof of the potential value of this kind of bioinformatics tool development for genomics and immunology.

In sum, we demonstrate that during T and B lymphocyte activation there are three classes of genes changing as a function of time: alternatively spliced and differentially expressed (AS+DE), constitutively expressed and alternatively spliced (AS+CE) and differentially expressed but not alternatively spliced (DE). We show that some canonical pathways are populated by only one class of genes, while other pathways, such as purine metabolism and T cell receptor signaling, are populated by all three classes of detected genes and these are changing in time. Our conclusion is that differential gene expression and/or alternative splicing of specific gene members in functional molecular networks during lymphocyte activation contributes to the regulation of these events. Moreover, the potential importance of constitutively expressed genes has been consistently ignored by previous studies of gene expression profiling that have focused only on differential gene expression. Our results now make it clear that the function and impact of many constitutively expressed genes could be significantly altered by alternative splicing events during lymphocyte activation. These studies expand the current views of T and B lymphocyte activation by providing evidence for a large number of molecular networks populated as a function of time and activation by alternatively spliced genes.

## Materials and Methods

### Human Lymphocyte Isolation

We purified CD2^+^ T-lymphocytes and CD19^+^ B-lymphocytes from Ficoll-Hypaque density separated peripheral blood mononuclear cells (PBMC) of 10 normal human donors. Miltenyi MACS CD2^+^ and CD19^+^ micro-magnetic beads were used for the positive isolation of CD2^+^ T-cells and CD19^+^ B cells, respectively, using a MACS separator with LS columns (MACS, Miltenyi Biotec).

### *Ex Vivo* Lymphocyte Activation, RNA and Protein Extraction

Freshly isolated, resting CD2^+^ T-cells were resuspended in RPMI-1640 complete media and activated with CD3/CD28 Dynal (Invitrogen) beads (25 µl beads to 1×10^6^ T cells). Cultures were sampled at 24, 48 and 72 hrs. A subset of isolated, resting cells immediately stabilized by RNALater (Ambion) were used as the baseline comparison (T0). The CD3 antibody coated on the CD3/CD28 T Cell Expander is specific for the epsilon chain of human CD3, a subunit of the TCR complex. The CD28 antibody is specific for the human CD28 co-stimulatory molecule, which is the receptor for CD80 (B7-1) and CD86 (B7-2). Both antibodies are coupled to the same Dynabead, mimicking in vivo stimulation by antigen presenting cells as a model for alloimmune activation [55]-[57]. Freshly isolated CD19^+^ magnetic-bead purified resting B cells were resuspended in RPMI-1640 complete media to which anti-CD40 antibody (G28–5 anti-CD40mAb at 1 µg/ml) was added, followed by cross-linking with goat-anti-mouse anti-IgG1 (STAR81, 0.2 µg/ml). The cells were then cultured with rIL2 and rIL10 (100 ng/ml each; Biosource) to simulate the T-cell dependent B cell activation of an alloimmune response [12], [58]-[65]. The cells were harvested and total RNA was extracted at time 0, 24, 48 and 72 hours post activation using the *mir*Vana miRNA Isolation Kit (Ambion). The *mir*Vana protocol also allows for the isolation of the total proteome fraction, which was collected for each time point and subsequently used in the MudPIT proteomics (see later).

### Analysis of Cell Activation by Multi-Channel Parallel Flow Cytometry

#### Cell surface staining

Cells were resuspended in ice cold PBE as follows: B and T cells for cell surface staining: 2×10^6^ cells/mL, T cells for intracellular staining: 3×10^6^ cells/mL. Cy5, Cy5.5 and Cy7-APC were coupled to individual monoclonal antibodies and titrated antibody-dye reagents were combined into pre-made cocktails. Cell suspension was added to 96-well plates and incubated with antibody cocktails at room temperature in the dark for 20 minutes. Buffer was added to each well to dilute unbound antibodies and stained cells were analyzed with SurroScan™ cytometers (Pharmaceutical Product Development (PPD), Inc, Menlo Park; http://www.ppdi.com/services/labs/biomarker/surroscan.htm).

#### Intracellular staining

Cell surface staining steps are the same except on ice and with washes. Cells are fixed with 0.5% formaldehyde for 20 minutes at room temperature, then permeabilized with 0.2% Saponin/5% FCS. Cells are incubated with cytokine antibodies in 96 well plates at room temperature in the dark for 20 minutes, followed by two washes with 0.2% Saponin/5% FCS and resuspended in buffer for SurroScan analysis.

#### Cell surface marker data analysis

A total of 1160 sample assays were performed in parallel (T cells  = 62 assays ×18 samples representing 9 donors at T0 and T48), B cells  = 18 assays ×8 samples representing 8 donors all done at T48 of activation). Standard gates were used in 90% of sample assays, non-standard gates used in 10% sample assays. Invalid sample assays were only 0.4%. Data from invalid sample assays were excluded from statistical analysis. Paired comparisons were performed for stimulated vs. controls for T cell data. B cell analysis was performed against PPD's archived historical data for unstimulated B cells (**Tables S1 and S2**).

#### Summary statistics on T cell data

Variable statistical levels were designated S1, S2 and S3, where S1 represents primary independent variables, S2 represents biologically meaningful but redundant variables, and S3 represents biologically irrelevant variables that were not considered. Step-down p-value adjustment was used on S1 variables. The number of variables at each p-value level are cumulative (See **Table S8**).

### The GeneChip® Human Exon 1.0 ST Array Profiling

1.5 µg of total RNA per sample was converted into labeled cDNA using the GeneChip® WT Sense Target Labeling kit (Affymetrix). Labeled cDNA was hybridized to Affymetrix Human Exon 1.0 ST arrays comprised of 1,404,693 probe sets that interrogate the whole known human genome with 10 hybridizations performed on the HumanExon1.0ST array per time point analysis (control vs. activated): 80 total  = 10 donors at 0, 24, 48, and 72 hrs for both T and B cells). Data for mRNA transcript profiles were generated in the form of CEL files using standard protocols (http://www.affymetrix.com/). The CEL files obtained were analyzed using the XRAY software version 2.51 (Biotique; http://www.biotiquesystems.com/Products-Solutions/XRAY//XRAY) to determine both differential as well as alternative splicing profiles. The entire set of CEL files from this study are available as Series GSE14352 at the NCBI Gene Expression Omnibus (GEO) site.

### XRAY Analysis of Gene Transcripts with Differential Expression and Alternative Splicing Events

Data expression values were collected as individual CEL files for each donor from the 1.0ST Exon Arrays and were then normalized in XRAY (www.biotiquesystems.com) with full quantile normalization. The 10 individual donors and 10 individual whole exon arrays (1 per donor) at each time point were normalized as a single file in XRAY comprised of 10 individual CEL files. This was done for each of the 4 time points (0, 24, 48 and 72 hours). These normalized, individual CEL files were then analyzed in XRAY for changes as a function of time after T or B cell activation. The normalized signal value data for each gene represents the median signal of non-spliced exons for that gene based on all 10 individuals. There were no technical replicates done. In accordance with best statistical practices, a linear model with Gaussian error was used to identify individual expression differences and calculate error across the 10 CEL files for each gene.

The normalized probe scores were pre-processed with background correction, probe summarization, and filtering of invariant and non-expressed probe-sets. After pre-processing, the “Core” probe-sets (the probe-sets annotated by Affymetrix as based upon the highest quality of genomic annotation) were analyzed with Mixed Model Analysis of Variance (ANOVA) to identify differential alternative splicing and/or differential expression. The nested model is appropriate because data is not sampled in a truly randomized fashion as expression points are harvested in batches defined by hybridizations (or individual CEL files). The mixed model is used since CEL files are random factors. The data generated above are analyzed with Analysis of Variance (ANOVA) according to the linear model:
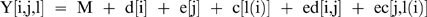
where M is a global mean, d(i) is the effect attributable to tissue state i, e(j) is the effect of exon j, and ec and ed are interaction effects. c, which is the hybridization (or chip) effect, is a random factor and all other factors are fixed. Note that the CEL file effect, c, is nested inside tissue state. Genes with significant D (tissue) effect are said to show significant tissue based gene expression difference. Genes with significant Exon-Tissue interaction (ED effect) are said to show signs of tissue specific alternative splicing (p-value<0.001). Thus, differential gene expression was identified as a significant) group effect (p-value<0.001) and alternative splicing as a significant tissue-probeset interaction (p-value<0.001). For each gene XRAY tests the probability of a ‘false-positive’ as a p-value >0.001. Since each of the individual gene tests are more or less independent tests and we are conducting a large number of tests, this uncorrected significance value could be misleading. The probability of finding a false-positive will grow as more genes are tested. To correct for this multiple testing challenge, we use the Benjamini and Hochberg False Discovery Rate (FDR) method, where the gene-level p-values are sorted in ascending order and then corrected. The sequential step-down procedure described above was used to calculate that the false discovery rate for this project is less than 1.00E+00 for differential alternative splicing and gene expression tests. The presence/absence of a gene in groups was assessed by deriving a p-value to test the null hypothesis that the group probe-set expression is not higher than the corresponding background probes. Rejection of the null hypothesis occurs when the p-value is less than the significance level of 0.001, in which case we infer that the gene is most likely expressed in the given state (for a detailed description of statistical calculations performed in XRAY, refer to **Methods S1**). In the final step, genes that passed the filtering criteria described here (i.e. expressed above background) and demonstrated statistical significance for differential gene expression and alternative splicing after passing the FDR-corrected ANOVA p-value<0.001 cut-off, were selected for further analysis.

### Sample Preparation and Deep-Sequencing of RNA

The RNA-Seq library preparation and sequencing were performed according to Illumina's protocol (www.Illumina.com). Specifically, 10 µg of total RNA, isolated from human T cells of a healthy donor and activated for 48 hours, was enriched for poly-A mRNA using oligo(dT)-magnetic beads. mRNA was fragmented to 100–300 bp-sized pieces using divalent cations at 95°C. RNA fragments were then copied into first strand cDNA using reverse transcriptase and random primers. This was followed by second strand cDNA synthesis using DNA Polymerase I and RNaseH. These cDNA fragments then were subjected to an end repair process, the addition of a single ‘A’ base, and then ligation of Illumina sequencing adapters. The ligated products were run on an agarose gel and a band was cut out of the gel corresponding to an insert length of approximately 200 bases in length. This product was then PCR amplified 12 cycles. The final sequencing library was created from gel purification of this PCR product and used directly for cluster generation and sequencing on an Illumina GAII system according manufacturer's instructions. Deep-sequencing was used to generate 60 bp-reads from 3 replicate lanes of a flowcell. The Genome Analyzer Pipeline Software (Pipeline) was used to perform the early data analysis of the sequencing run, including the image analysis, base calling, and alignment. Alignment was performed with Efficient Large-Scale Alignment of Nucleotide Databases (ELAND) which matches a large number of reads against the human genome with no more than two errors in the first 32 bases. Reads were mapped to the human genome (hg18, National Center of Biotechnology Information build 36.1).

### Ingenuity Pathway Analysis

Ingenuity Pathways are based on a constantly curated database of published literature on gene functions and interactions (https://analysis.ingenuity.com). The data represent molecules and pathways that are presently known and published. One of the outputs of Ingenuity analysis is functional pathways comprised of various numbers of candidate genes. Ingenuity also identifies “node genes” based on especially high degree of links to other genes in known pathways. The genes predicted by XRAY analysis to be significantly differentially expressed but not alternatively spliced (DE p<0.001 and AS p>0.001), significantly alternatively spliced and constitutively expressed (AS p<0.001 and DE p>0.001) and both significantly differentially expressed and alternatively spliced (DE p<0.001 and AS p<0.001) were used for Ingenuity Pathway Analysis. Each set of genes identified by the gene symbol and accompanied by either a fold-change for differential expression or alternative splicing (AS), or both (DE+AS), was loaded into the Ingenuity Pathway Analysis server to reveal biologically relevant interactions for activated T and B cells.

### Alternative Splice Validation by RT-PCR

Validation of alternative splicing was carried out by reverse transcribing 1 µg of total RNA from 8 donor samples at 0 and 48 hours to cDNA using the SuperScript First-Strand Synthesis System (Invitrogen) using random nonamer primers. PCR was carried out for 40 cycles using Taq Polymerase (Roche) per manufacturer's instructions with 1 µl of 40-fold diluted cDNA template. Primers were designed to adjacent constitutively expressed exons, in some cases spanning a few flanking exons. PCR products were separated on 2.5% high resolution Metaphor agarose gels (Lonza) stained with ethidium bromide for visualization. See **Table S5** for primer sequences.

### MudPIT Proteomics

We have used the Multi-dimensional Protein Identification Tool (MudPIT) protocol as previously described [67] using an LTQ XL mass spectrometer (ThermoFisher):

#### Sample preparation and data acquisition

Trizol was used for protein isolation. Total protein was denatured, alkylated, and trypsin digested as previously described[68]. 50 ug of digested protein sample (BCA, Pierce) was used for each experiment. Each sample was analyzed in four technical replicates. Mass spectrometry data were acquired using an LTQ LX linear ion trap mass spectrometer (ThermoFisher Scientific) interfaced in-line with 2D HPLC. The chromatography was setup as MudPIT [69] where discreet fractions from a front end strong cation exchange column were loaded onto and eluted from a reversed phase analytical column. Each experiment consisted of 14 discrete SCX fractions followed by a 130-minute linear reverse phase gradient from 0 to 50% Acetonitrile (AcN). Sample was introduced using nano-spray ESI at the flow rate of approximately 250 nl/min. The ESI voltage was held at 2.5 kV, and the capillary temperature was set to 200°C. Data sets were acquired in a data-dependant manner where each analytical full scan (MS, 200–2,000 *m/z* units range) was followed by three fragmentation scans (MS/MS) that targeted the three most abundant ions from the full scan. 40-micro second CID pulses of 35% intensity were used for precursor ion fragmentation. A default exclusion list (Xcalibur 2.0, ThermoFisher Scientific) of 180-second, 50 precursor ion members was used for data acquisition.

#### Proteomic data analysis

Raw data were searched against the EBI database (12/01/2006 release) supplemented with a decoy database where each entry of the original protein contains its reversed sequence. The database search was carried out using a PBS parallelized version of SEQUEST (v27)[70]. Search outcomes were post-processed and filtered using DTASelect [71] version 2.0 (in preparation). DTASelect 2.0 uses a quadratic discriminant analysis to dynamically adjust the XCorr and DeltaCN parameters to meet a required false positive rate (set to 0.05). Protein identifications were extracted and a measure of normalized amino acid coverage was used as label free quantification. The exact formula used to calculate relative protein abundance is as follows:
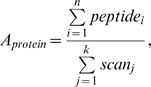
where *A_protein_* (protein abundance) is expressed as a ratio of the total redundant peptide identifications per protein to the total number of scans identified in the experiment. Each protein identification was annotated by GO association [72] (Revision 1.59, www.geneontology.org). Protein identifications across replicate experiments were pooled to represent a union for each category of 0 and 48-hour post activation. Proteins identified in two or more technical replicates per category were kept for further analysis. Relative protein abundance was compared between the 0 and the 48-hour post activation for proteins present in more than one category. A two-tailed, independent Student's t-test (pair-wise 0 vs. 48) was used for hypothesis testing (p-value cutoff of 0.05). Only the significant differentially expressed proteins were considered for functional analysis. Proteins identified in more than one technical replicate in a single category and not in any category were also considered for functional analysis as unique identifications.

## Supporting Information

Figure S1 Activation analysis of stimulated and resting CD4+ and CD8+ T cells. A) From top to bottom: Intensity plots showing levels of activation markers on CD4+ T cells and % of CD4+ T cells showing levels of activation markers. Intensity plots showing levels of chemokine receptors of CD4+ T cells and fraction of stimulated CD4+ T cells positive for chemokine receptors. Activation time point T48 or T0 hours is shown above the corresponding box plots. Cells stimulated for 48 hours (T48) are represented by clear box plots. Resting cells (T0) are represented by gray box plots. Target antigen is shown below the box plot, followed by the CYT-ID  =  a unique variable ID for each assay, ACT/CTR  =  mean ratio of stimulated vs. control cells, Effect Size  =  difference of means over SD, P-value  =  univariate P-value (color coded). Refer to panel D for explanation of the Bar Graph legends and P-value color codes. B) From top to bottom: Intensity plots showing levels of activation markers on CD8+ T cells and % of CD8+ T cells showing levels of activation markers. Intensity plots showing levels of chemokine receptors of CD8+ T cells and fraction of stimulated CD8+ T cells positive for chemokine receptors. Activation time point T48 or T0 hours is shown above the corresponding box plots. Cells stimulated for 48 hours (T48) are represented by clear box plots. Resting cells (T0) are represented by gray box plots. Target antigen is shown below the box plot, followed by the CYT-ID  =  a unique variable ID for each assay, ACT/CTR  =  mean ratio of stimulated vs. control cells, Effect Size  =  difference of means over SD, P-value  =  univariate P-value (color coded). Refer to panel D for explanation of the Bar Graph legends and P-value color codes. C) From top to bottom: CD127 (IL-7 receptor α chain) expression intensity for CD4+ and CD8+ T cells at T48 and T0 hours. Fraction of CD4 + T cells producing IFNγ, IL-10, IL-2, IL-4, and TNFα. Fraction of CD8+ T cells producing IFNγ, IL-10, IL-2, IL-4, and TNFα. Activation time point T48 or T0 hours is shown above the corresponding box plots. Cells stimulated for 48 hours (T48) are represented by clear box plots. Resting cells (T0) are represented by gray box plots. Target antigen is shown below the box plots. D) Graph legend. Cells stimulated for 48 hours (T48) are represented by clear box plots. Resting cells (T0) are represented by gray box plots. Variable statistics levels are represented by S1 (primary independent variables), and S2 (biologically meaningful but redundant variables). Step-down p-value adjustment was used on S1 variables and the number of variables at each p-value level are cumulative.
(1.98 MB TIF)

Figure S2 Activation analysis of stimulated B cells at 48 hours post activation. A) Intensity of stimulated B cells for activation markers CD150 (SLAM), CD25 (IL-2Rα), and CD69 (early activation marker). Only activated B cell data is shown. The staining of resting B cells from archived PPD data show intensities under 100 for this particular system using the SurroScan technology. B) Fraction of activated B cells positive for the activation markers. CYT-ID  =  unique variable ID for the assay, Mean  =  mean %, SD  =  standard deviation. C) Intensity of co-stimulatory molecule expression of CD54, CD80, CD86 for activated B cells. D) % of activated B cells that are positive for co-stimulatory molecules. E) Intensity and % of activated B cells that express CD21 and CD62L that are shed during B cell activation.
(0.96 MB TIF)

Table S1 CYT ID - Cytometry platform unique variable identifier CYT - Cytometry Cell Type - Top level classification of cell population Assay - The string consists of the target antigens separated by underscores and arranged by the channel number the reagent is measured on with SurroScan. Population - Describes the specific cell population. Names are based on the presence (p) or absences (n) of an individual antigen, e.g. CD3pCD8p represents CD3 positive CD8 positive T cells, i.e. CD8 T cells. A typical ASSAY may have 1 to 10 different populations as Property - Is the identifier of the type of statistic represented by the field VALUE such as COUNT (cells per uL) or INTENSITY (relative). Count and intensity results are typically generated for each POPULATION_NAME Count - Variable is an absolute cell count Ratio - Ratio of two population counts INTENSITY0 - Channel 0 intensity, the first antigen in assay INTENSITY1 - Channel 1 intensity, the second antigen in assay INTENSITY2 - Channel 2 intensity, the third antigen in assay p - Presence or positive stain of an antigen, e.g. CD3p represents CD3 positive T cells. n - Absences or negative stain of an antigen, e.g. CD3pCD4n represents CD3 positive and CD4 negative T cells. pn - Weaker positive in relative to stronger positive stain of an antigen in same assay, e.g. CD45RBpn and CD45RBp represent CD45RB dim and CD45RB bright population. SUM - Add up the counts of two or more daughter populations to obtain the counts of a parent population, e.g. the counts of CD4pCD45RApCD62Ln and CD4pCD45RApCD62Lp populations are added up to get the counts of CD4pCD45RAp_SUM population. Stat level - Output variables are classified into two statistical categories for comparative statistical analysis. S1 - primary variable statistic used in reduced variable set form primary analysis. S2 - secondary informative statistic biological useful represenation [−log(p-value)] - Negative logarithm (base 10) of the p-value for the given comparison Effect size - Mean difference between the groups/weighted SD, presented as an absolute value Mean ratios. Ratio of treated group to control group Mean - Mean for Cohort and TimeType SD - Standard Deviation %CV - % Coefficient of Variance (SD/Mean) AT - Activated T or B cells BC - Control T cells.
(0.16 MB XLS)

Table S2 CYT ID - Cytometry platform unique variable identifier CYT - Cytometry Cell Type - Top level classification of cell population Assay - The string consists of the target antigens separated by underscores and arranged by the channel number the reagent is measured on with SurroScan. Population - Describes the specific cell population. Names are based on the presence (p) or absences (n) of an individual antigen, e.g. CD3pCD8p represents CD3 positive CD8 positive T cells, i.e. CD8 T cells. A typical ASSAY may have 1 to 10 different populations Property - Is the identifier of the type of statistic represented by the field VALUE such as COUNT (cells per uL) or INTENSITY (relative). Count and intensity results are typically generated for each POPULATION_NAME Count - Variable is an absolute cell count Ratio - Ratio of two population counts INTENSITY0 - Channel 0 intensity, the first antigen in assay INTENSITY1 - Channel 1 intensity, the second antigen in assay INTENSITY2 - Channel 2 intensity, the third antigen in assay p - Presence or positive stain of an antigen, e.g. CD3p represents CD3 positive T cells. n - Absences or negative stain of an antigen, e.g. CD3pCD4n represents CD3 positive and CD4 negative T cells. pn - Weaker positive in relative to stronger positive stain of an antigen in same assay, e.g. CD45RBpn and CD45RBp represent CD45RB dim and CD45RB bright population. SUM - Add up the counts of two or more daughter populations to obtain the counts of a parent population, e.g. the counts of CD4pCD45RApCD62Ln and CD4pCD45RApCD62Lp populations are added up to get the counts of CD4pCD45RAp_SUM population. Stat level - Output variables are classified into two statistical categories for comparative statistical analysis. S1 - primary variable statistic used in reduced variable set form primary analysis. S2 - secondary informative statistic biological useful represen [−log(p-value)] - Negative logarithm (base 10) of the p-value for the given comparison Effect size - Mean difference between the groups/weighted SD, presented as an absolute value Mean ratios Ratio of treated group to control group Mean - Mean for Cohort and TimeType SD - Standard Deviation %CV - % Coefficient of Variance (SD/Mean) AT - Activated T or B cells BC - Control T cells
(0.39 MB XLS)

Table S3 (0.10 MB DOC)

Table S4 Detected known or putative splice factors in T cells at 48 hrs post activation.
(0.06 MB DOC)

Table S5 *XRAY T48/T0 exon call - direction of splicing change, activated vs control. *RNA Seq T48/T0 exon counts (3/3 samples)* - change in exon counts, activated vs control. *Functiona prediction - Proteins aligning or not aligning to the probeset. If the probe set is downregulated, then the probeset is expressed more highly in the control group. For example, (−)AA:1000(xxxxxxxx)→ 1200(xxxxxx) indicates that the shorter protein, with 1000 amino-acids is increased in the experimental group. If the probeset is upregulated,(+)AA indicates that the corresponding protein isoform is expressed more highly in the activated group. (−)alt-X-terminus indicates that the shorter isoform has a shorter terminus portion. (−)microRNA-target indicates loss of predicted microRNA binding sites. *uniprot-Ensembl feature predictions - Protein domains or functional regions increased (+) or decreased (−) in the experimental group. *Ensembl overlapping domains - reports overlaps between the identified probesets and the functional domains from Ensemble InterPro IDs that have either direct (indicated by (direct), complete alignment) or indirect (indicated by (indirect), partial overlap, typically occur in the gene introns) alignment between the probeset and domain genomic coordinates.
(0.10 MB XLS)

Table S6 (1.37 MB XLS)

Table S7 (0.05 MB XLS)

Table S8 (0.01 MB XLS)

Methods S1 (0.09 MB DOC)
