# Supplementary figures and images for: Genome-Wide Analysis of Immune Activation in Human T and B Cells Reveals Distinct Classes of Alternatively Spliced Genes
Source: PLoS One. 2009 Nov 19;4(11):e7906. doi: 10.1371/journal.pone.0007906 (PMC2775942; doi:10.1371/journal.pone.0007906)

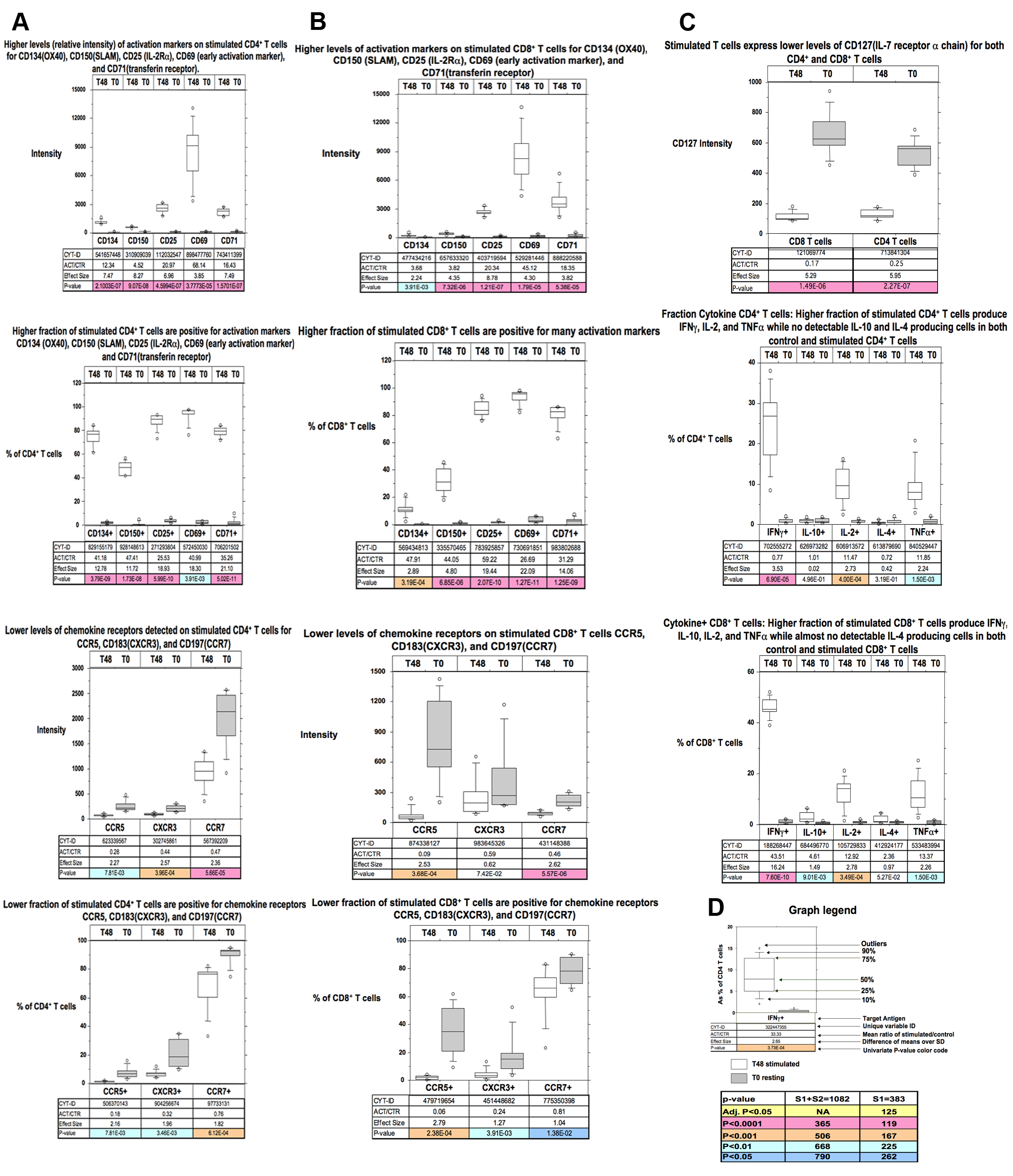

Supplement: Figure S1 — Activation analysis of stimulated and resting CD4+ and CD8+ T cells. A) From top to bottom: Intensity plots showing levels of activation markers on CD4+ T cells and % of CD4+ T cells showing levels of activation markers. Intensity plots showing levels of chemokine receptors of CD4+ T cells and fraction of stimulated CD4+ T cells positive for chemokine receptors. Activation time point T48 or T0 hours is shown above the corresponding box plots. Cells stimulated for 48 hours (T48) are represented by clear box plots. Resting cells (T0) are represented by gray box plots. Target antigen is shown below the box plot, followed by the CYT-ID = a unique variable ID for each assay, ACT/CTR = mean ratio of stimulated vs. control cells, Effect Size = difference of means over SD, P-value = univariate P-value (color coded). Refer to panel D for explanation of the Bar Graph legends and P-value color codes. B) From top to bottom: Intensity plots showing levels of activation markers on CD8+ T cells and % of CD8+ T cells showing levels of activation markers. Intensity plots showing levels of chemokine receptors of CD8+ T cells and fraction of stimulated CD8+ T cells positive for chemokine receptors. Activation time point T48 or T0 hours is shown above the corresponding box plots. Cells stimulated for 48 hours (T48) are represented by clear box plots. Resting cells (T0) are represented by gray box plots. Target antigen is shown below the box plot, followed by the CYT-ID = a unique variable ID for each assay, ACT/CTR = mean ratio of stimulated vs. control cells, Effect Size = difference of means over SD, P-value = univariate P-value (color coded). Refer to panel D for explanation of the Bar Graph legends and P-value color codes. C) From top to bottom: CD127 (IL-7 receptor α chain) expression intensity for CD4+ and CD8+ T cells at T48 and T0 hours. Fraction of CD4 + T cells producing IFNγ, IL-10, IL-2, IL-4, and TNFα. Fraction of CD8+ T cells producing IFNγ, IL-10, IL-2, IL-4, and TNFα. A [file pone.0007906.s001.tif]

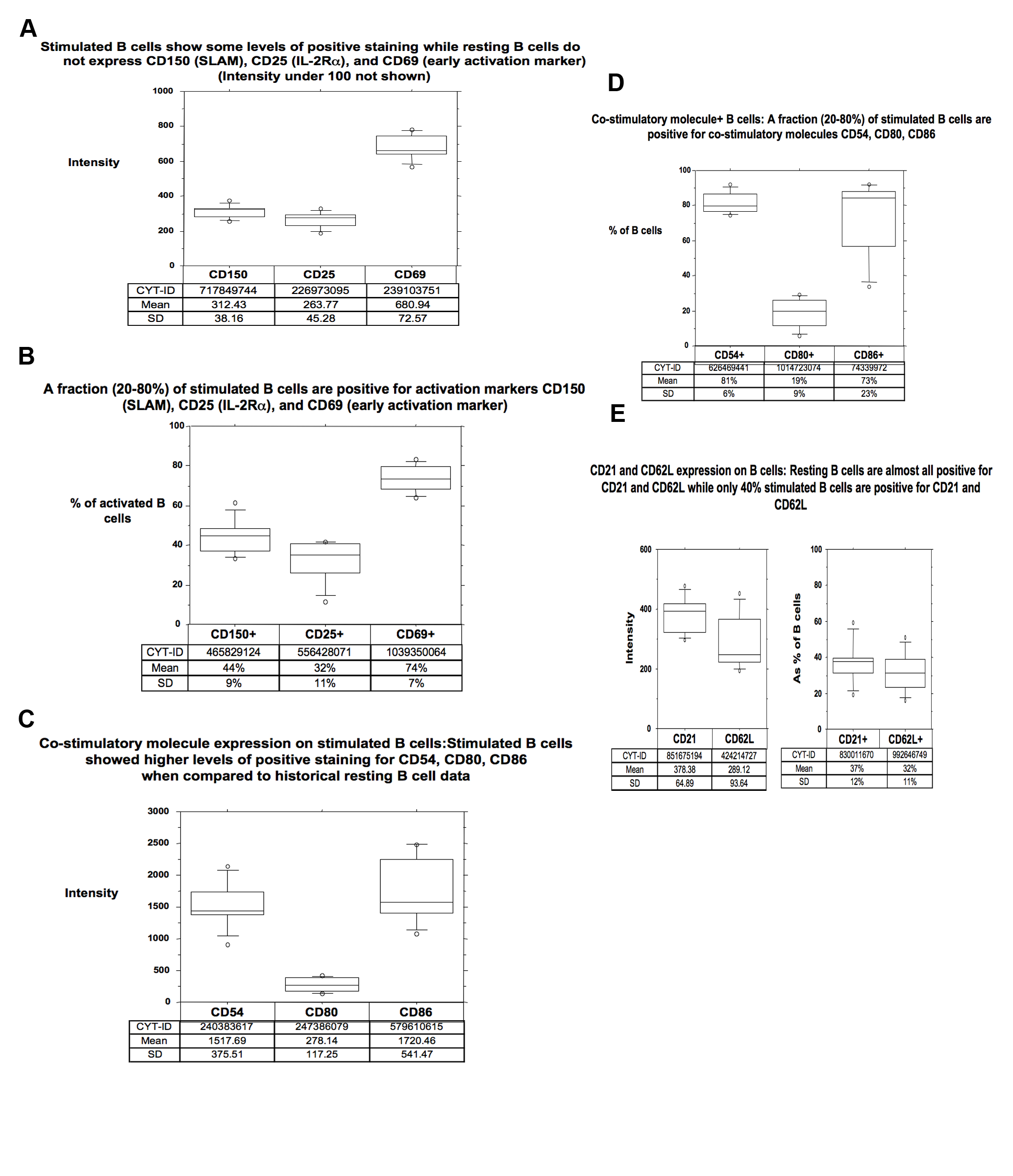

Supplement: Figure S2 — Activation analysis of stimulated B cells at 48 hours post activation. A) Intensity of stimulated B cells for activation markers CD150 (SLAM), CD25 (IL-2Rα), and CD69 (early activation marker). Only activated B cell data is shown. The staining of resting B cells from archived PPD data show intensities under 100 for this particular system using the SurroScan technology. B) Fraction of activated B cells positive for the activation markers. CYT-ID = unique variable ID for the assay, Mean = mean %, SD = standard deviation. C) Intensity of co-stimulatory molecule expression of CD54, CD80, CD86 for activated B cells. D) % of activated B cells that are positive for co-stimulatory molecules. E) Intensity and % of activated B cells that express CD21 and CD62L that are shed during B cell activation. (0.96 MB TIF) [file pone.0007906.s002.tif]
