# Supplementary material for: Genome-Wide Analysis of Immune Activation in Human T and B Cells Reveals Distinct Classes of Alternatively Spliced Genes
Source: PLoS One. 2009 Nov 19;4(11):e7906. doi: 10.1371/journal.pone.0007906 (PMC2775942; doi:10.1371/journal.pone.0007906)
Supplement: Table S3 — (0.10 MB DOC) [file pone.0007906.s005.doc]

| **Gene name** | **Function** | **Protein change** | **Functional change** | **Expression pattern** | **Ref** |
| --- | --- | --- | --- | --- | --- |
| FYN | PTK | in SH2 and kinase domains | FYNT better than FYNB at IL2 product. | T cells, FYNT increased on PMA stimul. of Jurkat | Rothrock, C, et al 2003, Weil, R et al 1999 |
| SYK | PTK | deletion of portion linker region | altered binding to ITAMs, less support of TCR-induced signal. | not determined in T cells | Rowley, R et al, 1995,Latour, S et al 1998 |
| ICAM1 | Adhesion (Ig) | altered number of immunoglobulin domains | different adhesion properties to LFA1 | some isoforms can differ in T cells stimulation with LPS | King, P. D. et al 1995 |
| PECAM1(CD31) | Adhesion (Ig) | altered cytoplasmic domain | not determined | increased cassette exon skipping on PMA stimulation of Jurkat, inhibitor of T cell activation | Wang, Y. & Sheibani, N. 2002, Wang, Y et al 2003 |
| CD44 | Adhesion, T cell homing | altered membrane proximity to extracellular domain | increase association with extracellular matrix polysac hyaluronan | increased cassette exon inclusion on antigen stimulation of naïve T cell or PMA stimulation of Jurkat | Ponta, H et al 2003 |
| CD45 | PTP, homeostasis, essential for activation of T cells | altered extracellular domain | change in dimerization capacity, phosphatase activity | increased cassette exon skipping on antigen stimul. Of resting T cell, attenuate TCR signaling | Lynch, K. W. & Weiss, A 2000. |
| CTLA4 | Cell-surface receptor, inhibition of T cell activation | change in inclusion of transmembrane domain | transmembrane vs. soluble | Increased inclusion of cassette exon(transmemb) on naïve T cell stimulation, prevent hyperstimulation | Oaks, M. K. et al 2000, Magistrelli, G. et al 1999 |
| TID1 | Apoptosis | altered carboxyl terminal | Pro-apoptotoc vs. anti-apoptotic | anti-apoptotic form increase on antigen stimulation of Th2 cells | Syken, J et al 2003 |
| CD95 | Apoptosis | change in inclusion of transmembrane domain | transmembrane vs. soluble receptor | Trasnsmembrane form is selectively increased on PBMCs stimulation in T cells | Liu, C et al 1995 |
| IL6 | Cytokine, T cell differentiation | del. Of a-heic. B & C | loss of interact w/IL6Rb, inhib of IL6 signal. | inhibition of IL-6 could block different of CD4+T to Th2 cells | Bihl, M. P. et al. 2002 |
| IL4 | Cytokine, T cell differentiation | omission of exon 2 that codes for active receptor complex | dominant negative signaling inhibitors | detected in all human peripheral blood mononuclear cells tested and in purified CD3+ T cells | Alms WJ, et al 1996 |
| IL2 | Cytokine, T cell differentiation | omission of exon that codes for active receptor complex | dominant negative signaling inhibitors | loss of stimulation for T cell proliferation | Tsytsikov et al 1996 |
| IL15 | Cytokine | alters translational start and leading sequence of peptide | different translational efficiency & targeting of peptide | not determined in T cells | Nishimura, H et al 1998 |
| IL4Ra | Cytokine Receptor | altered carboxyl terminal | transmembr. Vs. solub receptor | not determined in T cells | Kruse, S. et al 1999 |
| IL7Ra | Cytokine Receptor | change in inclusion of transmembrane domain | transmembr. Vs. solub receptor | Present in many T lineages, expression may increase in leukaemic cells | Goodwin, R. G. et al 1990 |
| PYK2 | PTK | deletion of portion of 1 of 2 proline-rich region | altered binding to var. prot. Partners | PYK2-H expressed by T and B cells. Longer version expressed in brain | Dikic, I. et al 1998 |
| IRAK1 | Ser/Threo Kinase associated with IL-1R | loss of 30 aa at terminus of kinase domaim | change in kinase activation and stability | Both forms expressed by thymocytes | Jensen, L. E. & Whitehead, A. S. 2001 |
| MyD88 | Intracel adaptor, association with IRAK4 | deletion of intermediate domain | change in ability to interact with IRAK4, loss of signaling. | Increased expression of smaller isoform on LPS stimulation of monocytes. | Burns, K. et al. 2003, Janssens, S et al 2002 |
| TIA-1 | pre-mRNA splicing and mRNA translation | Differ by 11 amino acids exclusive of the TIA-1a isoform | TIA-1b displays enhanced splicing stimul act compared with TIA-1a | funcion in apoptotic cell death adaptor, cellular response to metabolic stress & inflamation. | José M. Izquierdo and Juan Valcárcel, 2007 |
| SMAR1 | MAR-binding protein |  |  | changes of chrom. Struct. At TCRb locus dur early T develop. | Samit Chattopadhyay et al 2000 |
| CD79b | necessary for expression and function of the BCR | lacks exon 3 that encodes the extracellular Ig-like domain | reduced expression of BCR on the surface of B-CLL cells | might reflect the activation stage of the normal B cell from which B-CLL derives. | A. Alfarano et al 1999 |
| Pax-5 | transcription factor | proteins with only a partial DNA-binding domain | may be important regulator of transcription during B-cell maturation. | Alternatively Spliced during B-cell Development | Zwollo et al, 1997 |
| CD3zeta | TCR signaling |  | TCR retention and/or degradation in a pre-Golgi compartment. | expressed in T cells, granular lymphocytes and thymus | LK Clayton et al 1992 |
| TCRζ | TCR signaling | insertion of gluamate. between 1 & 2 amino term ITAMs within the cytoplasmic domain | TCR-mediated PLC activation | Surface expression of the T cell receptor complex is orders of magnitude less efficient in the absence of ζ | Atkinson T, et al 2003 |
| LyF-1/Ikaros | lymphocyte-specific DNA-binding protein | possess the N-terminal zinc finger domain | TdT promoter binding | interacts with gene promoters expressed during early stages of B- and T-cell development. | K Hahm 1994 |
| Sam68 | Links Signaling pathways to RNA processing | Removes RNA-binding KH domain, leaves protein-protein interaction domains intact | inhibit Cyclin D1, cell growth and RNA export | activated T cells | Lukong, K.E. & Richard, S, 2003 |
| VAV1 | Rho GEF and signaling adaptor molecule | Removes helix 1 of the DH domain which contains site of inhibitory loop interaction and much of GTPase binding site | abolishes GEF activity without altering adaptor function | activated T cells | Tybulewicz, V.L. et al 2005, Aghazadeh, B.,et al 2000. |
| MAP4K2 | Activator of MAP kinase pathways | In–frame removal of an exon encoding 44 aa of kinase domain | Loss of kinase activity, but leaves intact the C-terminal regulatory domain that is sufficient for MEKK1 activation | activated T cells | Chadee, DN et al 2002 |
| HMMR | Hyaluronan-binding protein involved in migration and signaling | Increased inclusion of an exon encoding15 highly charged amino acids | unknown | activated T cells | Assmann, V., et al 1998, Turley, E.A., et al 2002 |
| EIF4G2 | Regulator of translation | Deletion of 22 aa in Nterminus of protein | unknown | activated T cells | Lee, S.H. & McCormick, F. 2006 |
| LEF-1 | HMG-box transcription factor | In-frame deletion between regulatory phosphorylation sites and DNA binding domain | alter the ability of LEF-1 to induce structural rearrangements | activated T cells | Love, J.J. et al 1995 |
| HRB | Involved in mRNA export | In-frame deletion of 2 NPF repeats | Loss of interaction with Ehdomain proteins and Crm1 | activated T cells | Sanchez-Velar, N.,2004 |
| CUGBP2 | Inhibits translation of ARE-containing mRNAs, regulates splicing | Increased insertion of an exon that otherwise results in a PTC | Increased expression and activity | activated T cells | Mukhopadhyay, D.,et al 2003,Zhang, W.,et al 2002,Li, D., et al 2001 |
| AUF-1 | Promotes mRNA decay of ARE-containing messages | Insertion of exon 2, change from p37 to p40 form of protein | decreased AREmediated degradation | activated T cells | Lu, J.Y.,et al 2006, He, C. & Schneider, R.2006 |
| CLK2 | Dual-specificity kinase Phosphorylates splicing factors | Deletion of a 167 nt exon, leads to PTC | Loss of function and/or expression | activated T cells | Hanes, J et al 1994 |
| ILF3 | Regulator of Transcription and translation | Deletion of a 98 nt exon leads to a PTC | Increased protein expression and function | activated T cells at 72 hrs | Xu, Y.H.,et al 2003 |
| GATA3 | Transcription regulator | Removal of exon leads to PTC prior to DNA binding domain | Loss of DNA binding activity and/or expression | activated T cells, no AS detected in CD8+ T | Farrar, J.D. et al.2001 |
| HIF1a | Transcription regulator | Smaller isoform truncates ODD domain and lacks TAD. Functions as DN | Increased protein activity | activated T cells | Chun, Y.S.,et al 2002 |
| IRF1 | Transcription regulator | Smaller isoform lacks some of TAD and phosphoserine sites. | Altered transcriptional activation depending on cell type | activated T cells | Kim, E.J.,et al 2003 |
| FKBP | Multiple functions, induces cell cycle | Increase in isoform that encodes full length protein | Increase in protein expression & activity | activated CD8+ T cells | Aghdasi, B. et al.2001 |
| Erk1 | MAP kinase (S/T kinase) | Inhibits MEK1 interaction but retains dimerization and other interactions | Could function as a DN decreasing Erk function in cells | activated T cells | Robinson, F.L.,et al 2002 |
