# Supplementary material for: Genome-Wide Analysis of Immune Activation in Human T and B Cells Reveals Distinct Classes of Alternatively Spliced Genes
Source: PLoS One. 2009 Nov 19;4(11):e7906. doi: 10.1371/journal.pone.0007906 (PMC2775942; doi:10.1371/journal.pone.0007906)
Supplement: Table S4 — Detected known or putative splice factors in T cells at 48 hrs post activation. (0.06 MB DOC) [file pone.0007906.s006.doc]

| Gene Symbol  **Table S4: Detected known or putative splice factors in T cells at 48 hrs post activation** | Gene Annotation | Max fold change | Differential gene expression P value | Alternative exon usage P value |
| --- | --- | --- | --- | --- |
| SRPK1* | SFRS protein kinase 1 | 3.31 | 9.03E-06 | 1.00E+00 |
| SF3B3 | splicing factor 3b subunit 3 130kDa | 3.21 | 3.07E-06 | 1.00E+00 |
| SFRS1 | splicing factor arginine/serine-rich 1 (splicing factor 2 alternate splicing factor) | 2.51 | 5.23E-04 | 1.00E+00 |
| HNRPD | heterogeneous nuclear ribonucleoprotein D (AU-rich element RNA binding protein 1 37kDa) | 2.51 | 5.29E-04 | 1.00E+00 |
| SFRS4 | splicing factor arginine/serine-rich 4 | 2.21 | 2.49E-04 | 1.48E-01 |
| SF3B2 | splicing factor 3b subunit 2 145kDa | 2.11 | 2.75E-05 | 1.00E+00 |
| C4orf14 | chromosome 4 open reading frame 14 | 2.11 | 1.93E-03 | 1.74E-07 |
| U2AF1* | U2 small nuclear RNA auxiliary factor 1 | 2.01 | 3.19E-04 | 1.13E-10 |
| NDRG1 | N-myc downstream regulated gene 1 | 1.81 | 1.68E-02 | 9.72E-06 |
| U2AF2 | U2 small nuclear RNA auxiliary factor 2 | 1.71 | 8.47E-04 | 1.96E-02 |
| SF3A3* | splicing factor 3a subunit 3 60kDa | 1.71 | 2.60E-03 | 1.25E-07 |
| KHSRP | KH-type splicing regulatory protein (FUSE binding protein 2) | 1.51 | 3.50E-06 | 1.03E-12 |
| HNRPA1* | heterogeneous nuclear ribonucleoprotein A1 | 1.41 | 2.55E-05 | 1.00E+00 |
| HNRPLL | heterogeneous nuclear ribonucleoprotein L-like | 1.41 | 1.78E-02 | 1.16E-07 |
| PPRC1 | peroxisome proliferator-activated receptor gamma coactivator-related 1 | 1.41 | 1.28E-04 | 2.02E-03 |
| SFRS14 | splicing factor arginine/serine-rich 14 | 1.31 | 1.35E-02 | 1.02E-05 |
| SFRS3 | splicing factor arginine/serine-rich 3 | 1.21 | 3.08E-01 | 1.47E-05 |
| SFRS12 | splicing factor arginine/serine-rich 12 | 1.21 | 5.55E-01 | 3.43E-04 |
| SF3A1 | splicing factor 3a subunit 1 120kDa | 1.11 | 2.93E-01 | 2.07E-03 |
| SFRS6* | splicing factor arginine/serine-rich 6 | -1.01 | 4.19E-01 | 5.98E-08 |
| NDRG2 | NDRG family member 2 | -1.11 | 1.22E-04 | 1.29E-03 |
| SF1* | splicing factor 1 | -1.61 | 1.98E-04 | 1.00E+00 |
| SFRS5 | splicing factor arginine/serine-rich 5 | -1.71 | 2.20E-04 | 1.00E+00 |
| SFRS7* | splicing factor arginine/serine-rich 7 35kDa | -1.71 | 2.58E-02 | 1.66E-12 |
| CCNL1* | cyclin L1 | -1.81 | 3.78E-04 | 2.97E-02 |
| SFRS10** | splicing factor arginine/serine-rich 10 | -1.41 | 1.00E+00 | 2.84E-04 |
| CROP** | cisplatin resistance-associated overexpressed protein | 1.11 | 1.00E+00 | 2.69E-04 |
