# Supplementary material for: Genome-Wide Analysis of Immune Activation in Human T and B Cells Reveals Distinct Classes of Alternatively Spliced Genes
Source: PLoS One. 2009 Nov 19;4(11):e7906. doi: 10.1371/journal.pone.0007906 (PMC2775942; doi:10.1371/journal.pone.0007906)
Supplement: Methods S1 — (0.09 MB DOC) [file pone.0007906.s011.doc]

**An Analysis to Identify Differentially Expressed and Alternately Spliced Genes**

**Abstract**

In order to identify genes with differential gene expression and alternative splicing between the groups Control and Activated we study 20 hybridizations on the HumanExon1.0ST array. Contingency table analysis of the set of studied genes and a dataset of known pathways and gene classifications revealed that the set of alternatively spliced and expressed genes were found to be significantly overrepresented in groups of the GOMolFn, GOProcess, GOCellLoc, and Pathway classes (p<0.001).

**Methods For Project XRAY_TCL**

**Data Set**

The 20 input CEL files were analyzed with the Affymetrix HumanExon10ST array to identify genes that were significantly differentially expressed or displayed significant differential alternative splicing between the groups of interest. First, the input files were assigned to 2 groups as follows.

**Array Normalization**

The input files were normalized with full quantile normalization. For each input file, for each probe expression value, the i^th^ percentile probe value was replaced with the average of all i^th^ percentile points across all arrays. After sorting all the probe scores in the CEL files a master distribution is created and then the distribution of every CEL file is fit mathematically to the master.

**Low Level Data Handling**

Next, the 6553600 probes were manipulated into the analysis values as follows. Probes with GC count less than 6 and greater than 17 were excluded from the analysis. Non-expressed probes can cause tests for alternative splicing to find false positives (because they cause 'non-parallel' expression patterns across the gene). Hence, a one-sided t-test was used to identify probes that are significantly expressed above background probes of similar GC-content (i.e. we reject the null-hypothesis that the probe is not expressed above background if the p-value is less than 0.001. The background probes are defined in the file HumanExon10ST_antigenomic.bgp which is distributed by Affymetrix at www.affymetrix.com). Probe scores were then transformed by taking the Natural Logarithm of one plus the probe score.

Exon arrays do not use individual mis-match probes. Background probes are stratified by CG content and are defined in the HumanExon10ST_antigenomic.bgp file that can be downloaded from www.affymetrix.com. Each probe score was corrected for background by subtracting the median expression score of background probes of similar GC content from the same chip.

**Probe-set Expression Scores**

The Exon array contains 1404693 probe-sets (typically, but not always, groups of four probes).

Probe-set Expression Scores

The expression score for a probe-set was defined to be the median of its probe expression scores and probe-sets with fewer than 3 probes (that pass all of the tests defined above) are excluded from further analysis. Exon Array Probes are designed off of genomic sequence and hence the reliability of probes and probe-sets correspond to the quality of their parent genomic annotations. Probe-set reliability is ranked from more to less reliable as Core, Extended, or Full. For example 'Core' probe-sets include probe-sets that correspond to high quality genomic features like RefSeq (www.ncbi.nlm.nih.gov) or Ensembl (www.ensembl.org) transcripts while 'full' and 'extended' probe-sets match less reliable annotations like EST hits and gene prediction algorithms. For this analysis only 'Core' probe-sets were analyzed.

Removal of Non-expressed Probe-sets

For the same reasons mentioned above, it behooves us to remove non-expressed probe-sets from analysis. Probe-sets not significantly expressed above background (p-value > 0.001) are removed from analysis. Probe-set p-value is derived via Fisher p-value combination. Hence, accept a probe-set and reject the hypothesis that it has the same average expression as background if

-2 * Sum( Ln(probe DABG p-value) ) > crit. value

of Chi-Square(level 0.001, d.f. 2* Num_probes_In_Probe-set )

Filtering Invariant Probe-sets

Low-variance probe-sets are excluded from the analysis via a Chi-Square test. A probe-set is considered to be low-variance if its transformed variance is to the left of the 99.9 percent confidence interval of the Chi-Squared distribution with (N-1) degrees of freedom.

Transform = (N-1) * Probe-set Variance / (Probe-set Variance for gene) ~ Chi-Sq(N-1)

where N is the number of input CEL files, (N-1) is the degrees of freedom for the Chi-Squared distribution, and the 'probe-set variance for the gene' is the average of probe-set variances across the gene. Although, in practice, this method works well, it should be noted that the Chi-Square test of variance is usually applied to text a variance against a constant value and we are using it to test probe-set variance against a random variable (probe-set variance across gene) ; furthermore, the probe-set and probe-set across gene are not independent.

**Identification of Tissue Specific Gene Expression and Alternative Splicing**

Mixed Model, Nested Analysis of Variance was used to identify genes with tissue specific gene expression or alternative splicing. The nested model is appropriate because data is not sampled in a truly randomized fashion because expression points are harvested in batches defined by hybridizations (or individual CEL files). The mixed model is used since CEL files are random factors (i.e. we are not interested in the effect of CEL individual files since we are sampling from the many arrays that have been manufactured. In other words, if we were to redo the experiment we would use the same tissues and exons but we would use different CEL files).

The data generated above are analyzed with Analysis of Variance (ANOVA) according to the linear model

Y[i,j,l] = M + d[i] + e[j] + c[l(i)] + ed[i,j] + ec[j,l(i)]

where M is a global mean, d(i) is the effect attributable to tissue state i, e(j) is the effect of exon j, and ec and ed are interaction effects. c, which is the hybridization (or chip) effect, is a random factor and all other factors are fixed. Note that the CEL file effect, c, is nested inside tissue state. Genes with significant D (tissue) effect are said to show significant tissue based gene expression difference. Genes with significant Exon-Tissue interaction (ED effect) are said to show signs of tissue specific alternative splicing (p-value < 0.001).

Multiple Tests Correction

For each gene we test the probability of a 'false-positive' (or 'Type I Error') is 0.001. Since the individual gene tests are more or less independent and we are conducting a large number of tests, this significance value is misleading since the probability of finding a false-positive will grow as we test more genes. To correct for this we use the Benjamini and Hochberg False Discovery Rate (FDR) method where the gene-level p-values are sorted in ascending order and then corrected:

p_corrected = p_value * 1

for the largest p-value

p_corrected = p_value * (N/N-1)

for the second largest p-value, and

p_corrected = p_value * (N/N-2)

for the third largest p-value, etc... Where N is the total number of genes tested. It should be noted that while this method is regarded as a standard for expression analysis, it does not account for correlations between genes.

**Group Expression Level Filters to Reduce Alternative Splicing False Positives**

Large differences between tissue expression levels in a gene can cause a false positive for alternative splicing by introducing non-linear behavior that departs of the above model of expression. Specifially, the situation when exon expression of one group approaches background (or saturation) while other groups remain in the dynamic range causes "non-parallel" expression between the groups because the expression values in the dynamic range are free to vary while while samples near saturation or background are "dampened". This "non-parallel" behavior registers (falsely) as group specific alternative exon usage. We attempt to filter out these genes by looking large differences in p-values for tissue expression in the gene. In more precise terms the p-value is the likelihood of observing the gene-wide expression level under the null hypothesis that the tissue is not expressed above background so that a very low p-value means that the gene is most likely expressed in the given tissue. The p-value is assessed with a t-test between background probe-set expression and tissue probe-set expression.

Only One Group Expressed

Generate p-values for the 2 groups as above. Do not test genes for alternative splicing if only one tissue has exon expression (p-value less than 0.001).

**Input Data Quality Reports**

This section presents some quality metrics that can be calculated for each array. These include methods run before and after normalization.

**Before Normalization**

These views are generated with non-normalized, non-background corrected, untransformed probe-level data.


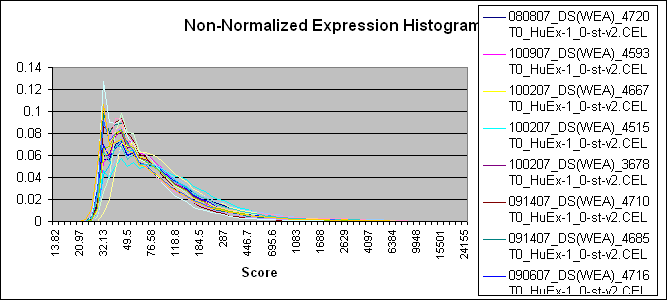
This graph shows the distribution of scores for each array. Each array has a line, the x axis represents score, and the y-axis represents the number of probes with score in the range divided by the total number of probes.


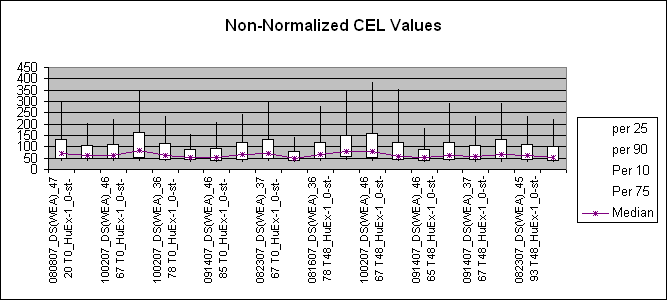
This graph shows the distribution of scores for each array. Each array is represented by a box plot where the middle (joined by lines to aid comparison) is the probe score median, the box top and bottom are the 25th and 75th percentiles of probe score, and the top and bottom lines are the 10th and 90th percentile of probe score.

**After Normalization**

These views are generated with normalized, non-background corrected, untransformed probe-level data. For details of the normalizations see the 'Normalization' section in 'Methods'.


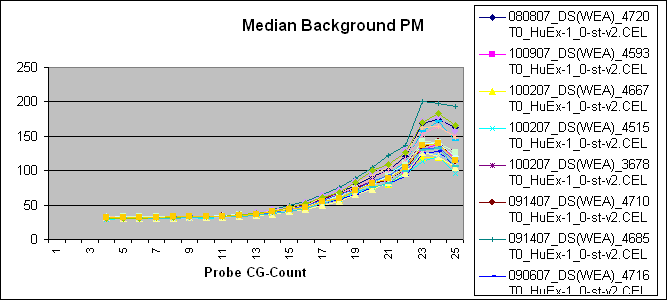
This graph shows the median normalized non-background corrected scores of probes designated as background. Each chip has a line, the x-axis represents GC count and the y axis is probe-score.


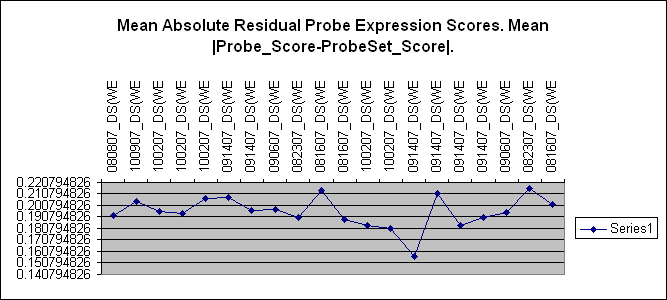
This graph shows the mean absolute difference between transformed, background-corrected (if specified), and normalized probe scores and summarized probe-set scores. The x-axis represents array and each array has a point on the graph. This is a quality score given by the ExACT package from Affymetrix.


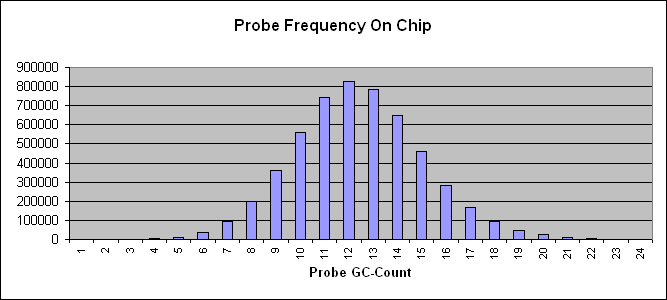
This graph shows a histogram where the x-axis is probe GC count and the y-axis is the frequency on the input arrays. This graph is, of course, the same before and after normalization.

**References**

Bolstad, B. M., Irizarry R. A., Astrand, M, and Speed, T. P. (2003). A Comparison of Normalization Methods for High Density Oligonucleotide Array Data Based on Bias and Variance. Bioinformatics 19,2,pp 185-193

Rafael. A. Irizarry, Benjamin M. Bolstad, Francois Collin, Leslie M. Cope, Bridget Hobbs and Terence P. Speed (2003), Summaries of Affymetrix GeneChip probe level data Nucleic Acids Research 31(4)

Burke, J. Biotique Systems (2006,2007). XRAY Software from Biotique Systems. www.biotiquesystems.com, www.orderxray.com.

Benjamini, Y. and Hochberg, Y. (1995). 'Controlling the False Discovery Rate: a Practical and Powerful Approach to Multiple Testing.' Journal of the Royal Statistical Society B, 57, 289-300.

Holm, S. (1979). 'A Simple Sequentially Rejective Bonferroni Test Procedure.' Scandinavian Journal of Statistics, 6, 65-70.

Irizarry, R.A., Hobbs B., Collin F., Beazer-Barclay, Y.D., Antonellis, K.UJ., Scherf, U., and Speed T.P. (2003). 'Exploration, Normalization, and Summaries of High Density Oligonucleotide Array Probe Level Data.' Biostatistics, 4, 249-264.
